# The role of myosin II in glioma invasion: A mathematical model

**DOI:** 10.1371/journal.pone.0171312

**Published:** 2017-02-06

**Authors:** Wanho Lee, Sookkyung Lim, Yangjin Kim

**Affiliations:** 1 National Institute for Mathematical Sciences, Daejeon, 34047, Republic of Korea; 2 Department of Mathematical Sciences, University of Cincinnati, Cincinnati, OH, 45221, United States of America; 3 Mathematical Biosciences Institute, Ohio State University, Columbus, OH, 43210, United States of America; 4 Department of Mathematics, Konkuk University, Seoul, 05029, Republic of Korea; University of Pécs Medical School, HUNGARY

## Abstract

Gliomas are malignant tumors that are commonly observed in primary brain cancer. Glioma cells migrate through a dense network of normal cells in microenvironment and spread long distances within brain. In this paper we present a two-dimensional multiscale model in which a glioma cell is surrounded by normal cells and its migration is controlled by cell-mechanical components in the microenvironment via the regulation of myosin II in response to chemoattractants. Our simulation results show that the myosin II plays a key role in the deformation of the cell nucleus as the glioma cell passes through the narrow intercellular space smaller than its nuclear diameter. We also demonstrate that the coordination of biochemical and mechanical components within the cell enables a glioma cell to take the mode of amoeboid migration. This study sheds lights on the understanding of glioma infiltration through the narrow intercellular spaces and may provide a potential approach for the development of anti-invasion strategies via the injection of chemoattractants for localization.

## Introduction

Glioblastoma multiforme (GBM) is the most common and aggressive type of primary brain tumors with the survival time of approximately one year from the time of diagnosis [1]. GBMs are characterized by the rapid proliferation and their infiltration into the surrounding normal brain tissue, resulting in inevitable and critical recurrence of a tumor even after conventional surgery [2].

An aggressive invasion of glioma cells into the surrounding tissue is one of the major reasons for the treatment failure leading to the poor survival rate. This is also due to the invisibility of individual migratory glioma cells even with current advanced technology and incomplete elimination of glioma cells by standard surgery [2]. Several biochemical factors such as EGF family [3] and remodeling of the extracellular matrix (ECM) may also contribute to the glioma cell infiltration in brain [4]. Furthermore, other types of cells such as microglia that are attracted to the tumor can secrete chemoattractants and they may contribute to the invasion of brain tumor [5].

**Fig 1:**
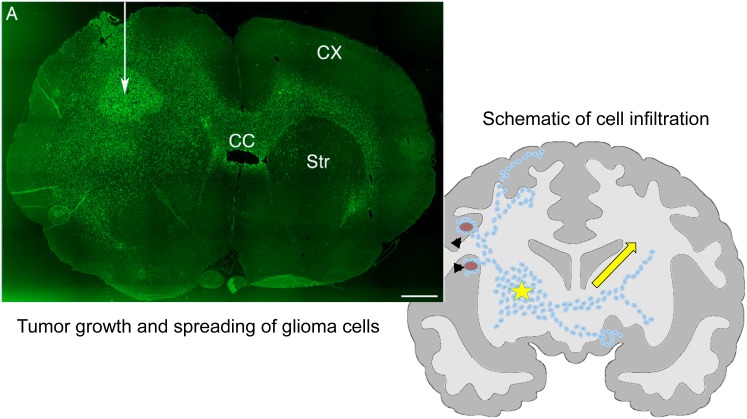
Experimental observation on cell infiltration in glioma models. (Left) Invasive Human glioma xenografts. Tumor has spread across the corpus callosum (CC) to the contralateral white matter located between straiatum (Str) and cortex (CX). Green = staining for human nuclear antigen to illustrate the location of human tumor cells in the rat background. White arrow = the location of the site of tumor inoculation. Reprinted from Beadle C, Assanah M, Monzo P, Vallee R, Rosenfield S, et al. (2008) The role of myosin II in glioma invasion of the brain. Mol Biol Cell 19: 3357-3368 [11] under a CC BY license, with permission from American Society for Cell Biology, original copyright 2008. (See S1 File) (Right) A schematic representation of diffuse infiltration of glioma cells. Arrowhead = blood vessels, asterisk = active tumor growth, arrow = tumor cells migrating along white matter tracks.

**Fig 2:**
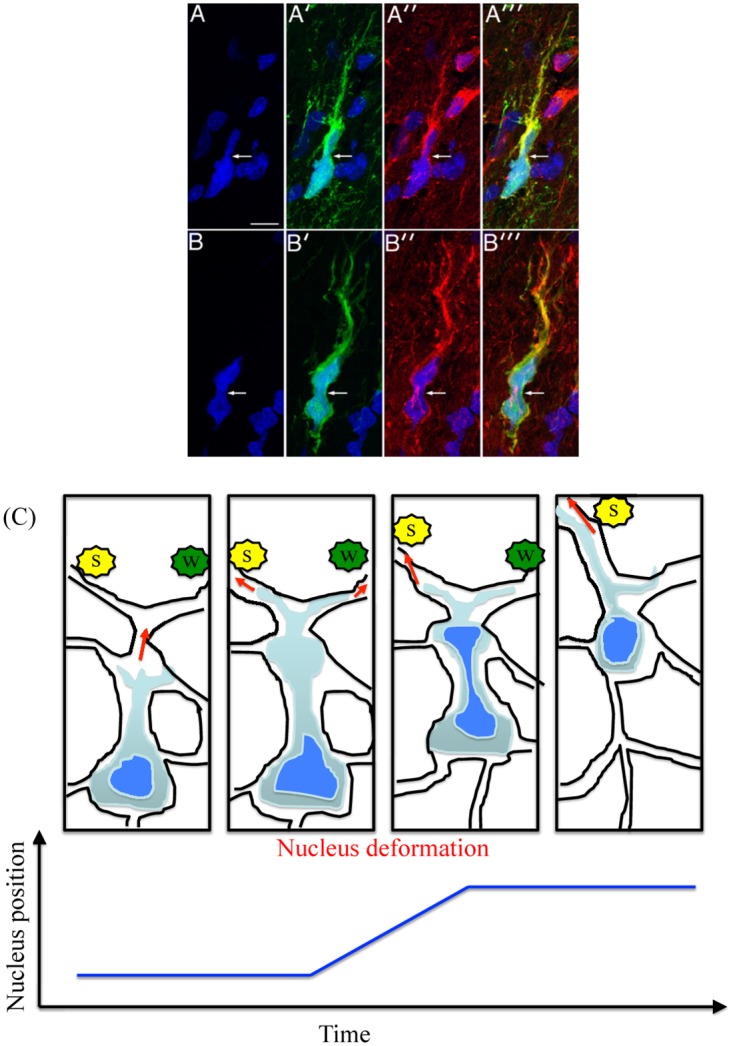
Nucleus deformation during cell migration in the glioma tissue. (A–A′′′, B–B′′′) Experimental observation of simultaneous cell body and nuclear deformation during migration through normal brain cells in a PDGF-driven glioma model [11]. (A, A′) A GFP-expressing human glioma cell (green) with staining of nuclear DAPI in (A) and GFP in (A′). (A′′) = strong red immunostaining for myosin IIA. (A′′′) = a merged image from (A), (A′), (A′′). (B, B′) Another infiltrating human glioma cell with DAPI and GFP staining. Strong red staining for myosin IIB was shown in (B′′). White arrows = focal deformation of the cell body, bar in (A) = 10 *μ*m. Reprinted from Beadle C, Assanah M, Monzo P, Vallee R, Rosenfield S, et al. (2008) The role of myosin II in glioma invasion of the brain. Mol Biol Cell 19: 3357-3368 [11] under a CC BY license, with permission from American Society for Cell Biology, original copyright 2008. See the main text for the detailed experimental setting. (C) A schematic of glioma cell migration through normal cells in the brain in response to biochemical signals [11]. Yellow star = a strong biochemical signal (s), green star = a weak biochemical signal (w). The bottom figure illustrates the nucleus position over time.

Glioma cells usually follow preferred migration routes, for example, the basal lamina of brain blood vessels or white matter tracts, see Fig 1 for the invasive behavior of glioma cells in brain tissue. This suggests that the migration of glioma cells may be regulated by specific substrates and structures in brain. The identification of common denominators of survived tumor cells after surgical resection may allow to develop new therapeutic approaches that target invasive cells [4, 6, 7] and hence improve clinical outcomes. Although infiltrative growth patterns of most glial tumors were observed about 70 years ago [8], there have not been effective therapeutic methods of eradicating the invading glioma cells yet. Glioma cells hold a remarkable capacity to infiltrate the brain and can migrate long distances from the primary tumor, creating huge challenges for complete surgical resection [9]. In addition, how glioma cells interact with the complex microenvironment is not completely understood. Cell migration through the dense network of normal cells is a complicated process that involves actin-myosin dynamics and complex signaling networks. The infiltrating glioma cells go through complicated processes including branching at its distal end (leading process), the forward movement of the centrosome and its associated microtubules (the dilatation [10]), the deformation of the nucleus, and the contraction of acto-myosin II at the rear of the cell, resulting in the saltatory forward movement. See Fig 2 for cell movement processes.

It is well known that tumor microenvironment affects tumor growth, invasion, and metastasis [1]. Glioma cell invasion depends on several microenvironmental factors such as chemotaxis, haptotaxis, and cell-cell adhesion [4]. The switching mechanism between cell proliferation and migration may depend on many signaling pathways, for example, miR-451-AMPK-mTOR [12, 13]. This miR-451-mediated go-or-grow behavior and growth patterns in response to fluctuating glucose were studied in [14, 15] using mathematical models. Recently, new anti-invasion strategies [16–19] were suggested, that is, localization of glioma cells after surgical resection of the primary tumor using the miR-451-AMPK core control system and injection of chemoattractants and glucose. Recently, Beadle *et al.* [11] showed that a glioma cell uses the ATP-dependent motor protein and myosin II, and the cell deforms not only its membrane but also its nucleus for migration through normal cells in the brain. Furthermore, mechanical stress and stiffness play a key role in regulation of overall tumor growth [20], cell migration [21], and metastatic potential in many cancer cell lines [22] including glioma [15, 16] and breast cancer [23]. Mechanical cell deformation was investigated in the studies of effects of microfluidic channel geometry and cell deformation on leukocyte rolling system [24, 25], amoeboid cell migration [26], dynamics of tumor cells in circulation [27], effect of matrix geometry on optimal cancer cell migration and interventions [28], and capsules and biological cells [29]. Cell migration through a confined ECM was also studied in [21].

In this work, we consider a two-stage cycle, elongation and retraction, for glioma cell migration through a dense network of normal glial cells in the tumor microenvironment. During the elongation step, the front part of the glioma cell protrudes while adhering the rear part of the cell to the substrate, and squeezes both the cell membrane and nucleus through the myosin II dynamics to infiltrate the intercellular gap between glial cells. Once the cell is fully elongated, the retraction step begins by releasing the rear adhesion sites and forming new adhesion sites at the front, pulling the back of the cell. Comparisons of experimental data with our computational results suggest that the regulation of myosin II is a fundamental feature in the deformation of the cell body and nucleus. Our findings also have important implications for glioma infiltration processes. In particular, the results suggest that microenvironmental factors such as chemoattractants and biomechanical structures of neighboring normal cells would permit early fate decisions to lead to patient-specific infiltration distributions. The methods can be generalized to other cancers because cell migration for resources (e.g. nutrient) is fundamental to tumor metastasis.

## Materials and methods

### Experimental setting

In the experiment [11], U251 human glioma cell lines and C6-GFP rat glioma [9] were cultured in mixture of Dulbecco’s modified Eagle’s medium and F-12 nutrient. These were used in *in vitro* and *in vivo* studies. The isolated glioblastoma cells were infected with a GFP-expressing lentivirus, enabling to track individual glioma cells at the infiltrating margin [11]. For human xenograft and immunohistochemical analysis, cultures of glioma cells were prepared from a surgical resection of a human glioblastoma and 10^5^ cells were infected with GFP-expressing lentivirus and transplanted into the subcortical area of white matter in adult nude rats [11]. After sacrifice, the 10*μm*-thick sections were stained with DAPI to stain the nuclei and with antibodies against GFP, myosin IIA, and IIB [11].

### Mathematical model of cell-mechanics

**Fig 3:**
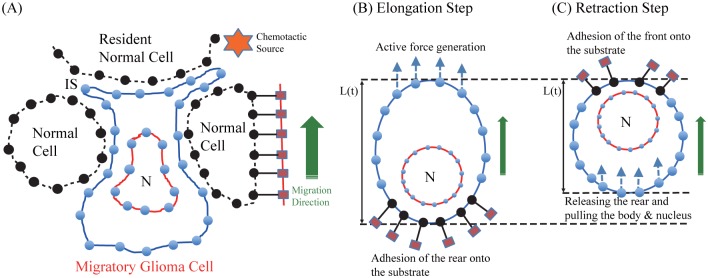
Discretized schematics of a mathematical model. (A) A schematic of glioma cell infiltration through narrow intercellular space (IS) between normal resident glial cells in the direction (green arrow) of a chemotactic source (red star; *). A glioma cell is described by two elastic closed curves, representing the outer cell boundary (blue solid lines) and the boundary of the nucleus (red solid lines; ‘N’). The boundaries consist of elastic springs connected by nodes. Resident normal cells (black dashed lines) are treated in a similar fashion but without the nucleus. Normal cells are assumed to be relaxed and passively respond to biochemical and biomechanical stimuli, in other words, cells are tethered in the tissue (red lines and box). (B) During the elongation step, active force is generated at the front of the cell body and adhesion onto the substrate is formed at the rear of the cell. (C) In the retraction step, the glioma cell pulls both cell body and nucleus forward by forming attachment at the front and releasing the attachment of the rear edge.

In our model, we consider two types of cells, a tumor (glioma) cell and normal cells, which are immersed in a viscous fluid. A glioma cell migrates through the dense ECM and other cells within a brain. This glioma cell is represented by two elastic closed loops, an inner loop corresponding to the nucleus of the cell and an outer loop corresponding to the cell basal membrane. Unlike the glioma cell, each normal cell is represented by an elastic curve, corresponding to the basal membrane only that is tethered to the tissue, see Fig 3A. The immersed boundary (IB) method is used to simulate the interaction between the viscous fluid and the elastic cells [30].

Let ***X***(*s*, *t*) = ***X***^*Gm*^(*s*, *t*)⋃***X***^*Gn*^(*s*, *t*)⋃***X***^*N*^(*s*, *t*) be the configuration of cells at any time *t*, where *s* is a moving curvilinear coordinate system. Here, ***X***^*Gm*^ and ***X***^*Gn*^ represent the membrane and the nucleus of a glioma cell, respectively. ***X***^*N*^ represents the membrane of normal cells. Note that nuclei of normal cells are not considered in this work. The coupled system of equations of motion is given as follows:

$$\begin{array}{c} \rho (\frac{\partial u}{\partial t}+u\cdot \nabla u)=-\nabla p+\mu \Delta u+f, \end{array}$$ (1)

$$\begin{array}{c} \nabla \cdot u=0, \end{array}$$ (2)

$$\begin{array}{c} f(x,t)=\int F(s,t)\delta (x-X(s,t))ds, \end{array}$$ (3)

$$\begin{array}{c} \frac{\partial X(s,t)}{\partial t}=\int u\left(x,t\right)\delta \left(x-X\left(s,t\right)\right)dx, \end{array}$$ (4)

$$\begin{array}{c} F=F_{\text{e}}^{Gm}+F_{\text{e}}^{Gn}+F_{\text{e}}^{N}+F_{\text{t}}^{Gm}+F_{\text{t}}^{N}+F_{\text{a}}^{Gm}. \end{array}$$ (5)

Fluid Eqs (1) and (2) are the incompressible Navier-Stokes equations, where ***u***(***x***, *t*) is the fluid velocity, *p*(***x***, *t*) is the fluid pressure, and ***f***(***x***, *t*) is the applied fluid force density defined on a fixed Cartesian coordinate system, where ***x*** = (*x*_1_, *x*_2_). The constant parameters *ρ* and *μ* are the fluid density and the fluid viscosity, respectively.

Eqs (3) and (4) are the interaction equations that connect the fluid variables and the immersed boundary variables by the two-dimensional Dirac delta function *δ*, where ***F***(*s*, *t*) is the boundary force density which will be described in detail below. Eq (3) describes the relationship between the two corresponding force densities ***f***(***x***, *t*) and ***F***(*s*, *t*), and Eq (4) is the no-slip condition which means that the structure moves at the local fluid velocity.

Eq (5) is the immersed boundary equation. Here ***F***(*s*, *t*) is the force density which acts on the fluid by the immersed boundary. There are six contributions to the force density function ***F***: the elastic force density $F_{\text{e}}^{Gm}$ from the glioma cell membrane, the elastic force density $F_{\text{e}}^{Gn}$ from the nucleus inside the glioma cell, the elastic force density $F_{\text{e}}^{N}$ from the normal cell membrane, the tethered force density $F_{\text{t}}^{Gm}$ acting on the part of the glioma cell membrane, the tethered force density $F_{\text{t}}^{N}$ from the normal cell membrane, and the active force density $F_{\text{a}}^{Gm}$ of the glioma cell membrane for migration. These force densities are obtained in the following

$$\begin{array}{c} F_{\text{e}}^{Gm}=\frac{\partial E^{Gm}}{\partial X^{Gm}}, \end{array}$$ (6)

$$\begin{array}{c} F_{\text{e}}^{Gn}=\frac{\partial E^{Gn}}{\partial X^{Gn}}, \end{array}$$ (7)

$$\begin{array}{c} F_{\text{e}}^{N}=\frac{\partial E^{N}}{\partial X^{N}}, \end{array}$$ (8)

$$\begin{array}{c} F_{\text{t}}^{Gm}=\{\begin{array}{cc} c_{\text{t}}^{Gm}\left(Z_{r}^{Gm}-X_{r}^{Gm}\right), & \text{during}\;\text{elongation}\;\text{step}\\ c_{\text{t}}^{Gm}\left(Z_{f}^{Gm}-X_{f}^{Gm}\right), & \text{during}\;\text{retraction}\;\text{step} \end{array} \end{array}$$ (9)

$$\begin{array}{c} F_{\text{t}}^{N}=c_{\text{t}}^{N}\left(Z^{N}-X^{N}\right), \end{array}$$ (10)

$$\begin{array}{c} F_{\text{a}}^{Gm}=\{\begin{array}{cc} c_{\text{a}}\vec{d}, & \text{during}\;\text{elongation}\;\text{step}\\ 0 & \text{during}\;\text{retraction}\;\text{step} \end{array} \end{array}$$ (11)

In Eqs (6)–(8), *E*^*Gm*^, *E*^*Gn*^ and *E*^*N*^ are the elastic stretching energy functionals with respect to the configurations ***X***^*Gm*^, ***X***^*Gn*^ and ***X***^*N*^, respectively. Here, the elastic stretching energy functional *E* for a given configuration $\bar{X}$ is defined by

$$\begin{array}{c} E(\bar{X})=\frac{c}{2}\int {\left(|\frac{\partial \bar{X}}{\partial s}|\right)}^{2}ds, \end{array}$$ (12)

where *c* is the stiffness coefficient for the elastic boundary. Note that *c* may be changed into $c_{\text{e}}^{Gm}$, $c_{\text{e}}^{Gn}$, and $c_{\text{e}}^{N}$ in Eqs (6)–(8), respectively. The elastic force density can be obtained from minus gradient of the energy functional, *i.e.,* $-\frac{\partial E}{\partial \bar{X}}$, and is therefore described by

$$\begin{array}{c} \bar{F}\left(\bar{X}\right)=\frac{\partial }{\partial s}\left(T\cdot \tau \right), \end{array}$$ (13)

where

$$\begin{array}{c} T=c|\frac{\partial \bar{X}}{\partial s}|, \end{array}$$ (14)

$$\begin{array}{c} \tau =\frac{\partial \bar{X}}{\partial s}/|\frac{\partial \bar{X}}{\partial s}|, \end{array}$$ (15)

where *T* is the tension derived from Hooke’s law and ***τ*** is the unit tangent vector to the elastic boundary.

**Fig 4:**
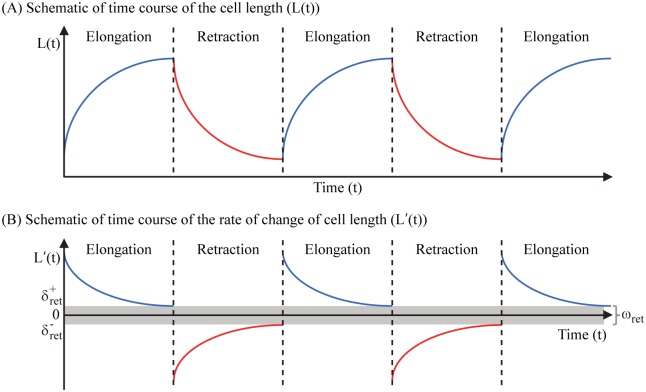
Schematics of the deformation of cell body during elongation and retraction steps. Schematic of changes in the cell body length (*L*(*t*) in (A)) and the rate of change of cell length (*L*′(*t*) in (B)).

In Eq (9), $Z_{r}^{Gm}\left(t\right)$ is the reference configuration of the surrounding tissue for the attachment of the *rear* part of the moving glioma cell (denoted by $X_{r}^{Gm}$) during the elongation step [31]. During this step, cross-linked networks at the front of the cell drive cell movement by polymerizing the actin and pushing the cell membrane [31], see Fig 3B. In a similar fashion, $Z_{f}^{Gm}\left(t\right)$ is the reference configuration of the surrounding tissue for the attachment of the *front* part of the moving glioma cell (denoted by $X_{f}^{Gm}$) during the retraction step [31]. During this step, biochemical attachment of the rear part of the cell body to the surrounding substrate is removed and the cell pulls its cell body including nucleus by using the attachment of the front part of the cell body, see Fig 3C. Note that the cell body length *L*(*t*) is defined by the longest distance between the front and rear parts of the cell membrane, see Fig 3B and 3C. The nucleus length *N*(*t*) is also defined in a similar way. Fig 4 shows schematics of changes in the cell body length (*L*(*t*) in Fig 4A) and the rate of change of cell length (*L*′(*t*) in Fig 4B) during elongation and retraction steps. During the elongation step shown in Fig 3B, the rate (*L*′(*t*) > 0) starts from the maximum value and then decreases to the limit value, so called retention rate $\delta_{ret}^{+}$ ($\delta_{ret}^{+}>0$). This leads to a steady increase in the cell body length (blue solid curve in Fig 4A). The retraction step shown in Fig 3C leads to an increase in *L*′(*t*) to the retention rate $\delta_{ret}^{-}$ from the minimum value (red solid curves). This results in a steady decrease in the cell body length (red solid curve in Fig 4A). Here, *L*′(*t*) < 0 and $\delta_{ret}^{-}<0$. The width ($\omega_{ret}=\delta_{ret}^{+}-\delta_{ret}^{-}$) of the retention strip (box in gray) depends on the cell’s biomechanical response to complex microenvironment such as narrow gaps between normal glial cells. In Eq (10), $Z^{N}$ is the reference configuration of the surrounding tissue for resident normal cells. In Eqs (9) and (10), $c_{\text{t}}^{Gm}$ and $c_{\text{t}}^{N}$ are the stiffness coefficients for the tethered glioma cell membrane and normal cells, respectively.

In Eq (11), *c*_a_ is the active force constant and $\vec{d}$ is the unit vector in the moving direction. It has been known that a cell can sense the microenvironment and decide the migration direction in response to many biochemical signals. We assume that in a given microenvironment the migration direction $\vec{d}$ is a function of the gradient ∇*C* of chemoattractants *C*(***x***, *t*), which will be described in detail below, *i.e.,* we assume that a cell migrates in the direction of the highest gradient within a given disk *B*(***x***; *r*_*s*_) with a sensing radius $R_{s}^{c}$:

$$\begin{array}{c} \vec{d}\left(x\right)=\frac{1}{\left|B(x;R_{s}^{c})\right|}\int_{B(x;R_{s}^{c})}\nabla C\left(y,t\right)dy. \end{array}$$ (16)

Let *Ω* = [0, 0.1] × [0, 0.1](*mm* × *mm*) be the computational domain of the model in a two-dimensional space. The parameter values and the reference values are given in Table 1. To solve the above equations on *Ω* numerically, we discretize the equations in space and time using a finite difference scheme. In particular, we use FFTs (Fast Fourier Transforms) to solve the discretized Navier-Stokes equations with periodic boundary conditions. For a detailed description of the numerical scheme, the reader is referred to [30, 32].

**Table 1:** Parameters used in this work. (tw = estimated in this work.)

Par	Description	Value	References
**IB method**			
*l* × *l*	fluid domain	0.1 *mm* × 0.1 *mm*	
*N* × *N*	grid size	512 × 512	
*μ*	fluid viscosity	2.7 *g*/(*cm* ⋅ *s*)	[15]
*ρ*	fluid density	1.35 *g*/*cm*^3^	[33, 34]
△*t*	time step	0.004*s*	
*r*^*G*^	radius of a glioma cell	5 *μm*	[15, 16, 18]
*r*^*N*^	radius of a normal cell	8 *μm*	[15, 16, 18]
$c_{\text{e}}^{Gm}$	elastic stiffness of a glioma cell	3.8 × 10^−5^ *g* ⋅ *cm*/*s*^2^	tw
$c_{\text{e}}^{Gn,b}$	basal elastic stiffness of the nucleus	3.8 × 10^−5^ *g* ⋅ *cm*/*s*^2^	tw
$c_{\text{e}}^{N}$	elastic stiffness of a normal cell	0.0023 *g* ⋅ *cm*/*s*^2^	tw
$c_{\text{t}}^{Gm}$	tethered stiffness of a glioma cell membrane	1500 *g*/*cm* ⋅ *s*^2^	tw
$c_{\text{t}}^{N}$	tethered stiffness of a normal cell	400 *g*/*cm* ⋅ *s*^2^	tw
*χ*	the basal active force strength	0.7	tw
*λ*_*s*_	chemotactic parameter	20 (*s*^2^/*g*)^2^	tw
**Myosin II model**			
*R*_*s*_	sensing radius for (mechanical pressure)	4.69 *μm*	[4]
$R_{s}^{c}$	sensing radius (chemotaxis)	0.4 *μm*	[4]
*k*_1_	association rate (bound myosin II)	0.002 *μM*^−1^ *s*^−1^	[35], tw
*k*_−1_	dissociation rate (bound myosin II)	0.0001 *s*^−1^	[35], tw
[*m*_*T*_]	total myosin II concentration	1 *μM*	[36]
*k*_*p*_	myosin-pressure sensitivity (wild type)	0.9 *μM* ⋅ *s*^2^/(*g* ⋅ *cm*)	tw
	myosin-pressure sensitivity (MYOII-KD)	0.045 *μM* ⋅ *s*^2^/(*g* ⋅ *cm*)	tw
*n*	Hill coefficient (myosin reaction)	10	tw
*K*_*mb*_	reciprocal of the critical threshold	1.8 *μM*^−1^	tw
*k*_*s*_	nucleus-myosin constant	5	tw
$k_{s}^{min}$	nucleus-myosin constant	0.1	tw
**Reaction-diffusion (chemoattractant)**			
*D*_*C*_	diffusion coefficient	2.15 × 10^−6^ *cm*^2^/*s*	[7, 37, 38], tw
$\lambda_{in}^{C}$	chemoattractant source	8.2 × 10^−1^ *g*/(*cm*^3^.*s*)	[38]
*μ*_*C*_	decay rate	1.0 × 10^−6^ *s*^−1^	[7, 38, 39], tw
**Therapeutic drugs**			
*I*_*B*_	injection rate of blebbistatin	5 × 10^−2^ *μM*/*s*	tw
*μ*_*B*_	decay rate of blebbistatin	5.13 × 10^−4^ *s*^−1^	[18, 40, 41]
*α*	degradation rate of the bound myosin II by blebbistatin	1.0 × 10^−4^ *s*^−1^(*μM*)^−1^	tw
*μ*_*D*_	decay rate of drugs targeting actin-myosin association	5.13 × 10^−4^ *s*^−1^	[18, 40, 41]

### Dynamics of myosin II

A motor, myosin II, plays a significant role in glioma invasion *in vivo*, in which myosin II regulates the deformation of the nucleus as well as the membrane of glioma cells [11]. An actin filament network is a key component of the cytoskeleton of a cell and is considered to be one of the most important mechanical structures that are involved in cell migration [42]. The cell generates a protrusive force at the leading edge through polymerization of actin filaments and creates the contractile stress at the rear via acto-myosin binding [43]. Experimental evidences indicate that the cell stiffness is proportional to the F-actin density, and the elastic modulus of a cell may increase up to ten-fold from the rear of a lamellipod to its front [42]. In this work, we assume that the acto-myosin motors are activated by the formation of actin-myosin II network. To take the above into account, we consider the following chemical reactions within a cell [35]:

$$\begin{array}{c} m_{f}+a\overset{k_{1}}{\underset{k_{-1}}{⇌}}m_{b}, \end{array}$$ (17)

where *m*_*f*_, *a*, *m*_*b*_ are concentrations of free myosin II, actin filament, and bound myosin II, respectively, and *k*_1_ and *k*_−1_ are reaction rate constants. We assume that the total number [*m*_*T*_] of myosin II molecules, sum of free and bound myosin II molecules, remains constant, *i.e.,* [*m*_*T*_] = [*m*_*f*_] + [*m*_*b*_] = constant. Given the actin filament concentration [*a*], the chemical reactions Eq (17) above lead to the following differential equation for bound myosin II molecules:

$$\begin{array}{c} \frac{d[m_{b}]}{dt}=k_{1}\left[m_{f}\right]\left[a\right]-k_{-1}\left[m_{b}\right]=k_{1}\left[m_{T}\right]\left[a\right]-\left(k_{1}\left[a\right]+k_{-1}\right)\left[m_{b}\right]. \end{array}$$ (18)

Glioma invasion depends on microregional heterogeneity of the ECM and its modifications in tumoral brain [44]. Infiltrating glioma cells can sense the microenvironment and respond to bio-mechanical cues for different adaptations in cell migration [9, 11]. In order to translate the mechanical pressure in the microenvironment to the dynamics of actin filaments, we define sensing pressure, $p_{B}^{s}\left(x,t\right)$, at a given point ***x*** and time *t* as follows:

$$\begin{array}{c} p_{B}^{s}\left(x,t\right)=\frac{1}{\left|B(x,R_{s})\right|}\int_{B(x,R_{s})}p\left(y,t\right)dy, \end{array}$$ (19)

where *p*(***y***, *t*) is the microenvironmental pressure in Eq (1), and *B*(***x***, *R*_*s*_) is a disk with the *sensing radius* *R*_*s*_ centered at the given point ***x***. Note that the idealized microenvironment can be approximated by a disk *B*(***x***, *R*_*s*_) [4]. Then the average of the sensing pressure over the cell membrane, *p*^*s*^(*t*), is defined by,

$$\begin{array}{c} p^{s}\left(t\right)=\frac{1}{|X^{Gm}|}\int_{X^{Gm}}p_{B}^{s}\left(x,t\right)dx, \end{array}$$ (20)

and in what follows the concentration of actin filaments is defined by

$$\begin{array}{c} \left[a\right]=k_{p}p^{s}\left(t\right), \end{array}$$ (21)

where *k*_*p*_ is the myosin-pressure sensitivity.

Each migratory glioma cell is able to deform its nucleus using the motor, myosin II, while the cell migrates through densely packed other cells of the brain [10]. However, myosin II is not required to push the cytoplasm through either relatively larger transwell pores or brain parenchyma [11] because the action of actin filament alone is enough for the migration due to the flexibility of plasma membrane. This is also consistent with a recent study that the nuclear envelope is much stiffer than the plasma membrane [45]. Sakamoto *et al.* [35] showed that the modulus of contractile elasticity reaches its maximum in the middle part of the cell, which is responsible for generating the force to pull the cell body forward. This result is consistent with the experimental observation that acto-myosin contractile activity is localized in the cell body, immediately behind the protrusive region due to more myosin II activities [43]. Therefore, the stiffness of the nucleus and contractile force depend on the activity of myosin II. In our model, the stiffness of the nucleus, $c_{\text{e}}^{Gn}$, is given by

$$\begin{array}{c} c_{\text{e}}^{Gn}=c_{\text{e}}^{Gn,b}\times r_{\left[m_{b}\right]}, \end{array}$$ (22)

where $c_{\text{e}}^{Gn,b}$ is the basic reference stiffness of the nucleus when the myosin II is not activated and *r*_[*m*_*b*_]_ is the myosin II-dependent stiffening rate of the nucleus. Note that $c_{\text{e}}^{Gn}$ is used to evaluate the elastic force from the nucleus in Eq (7). Finally we employ a Hill-type function for the stiffening rate *r*_[*m*_*b*_]_ as a function of [*m*_*b*_] as follows:

$$\begin{array}{c} r_{\left[m_{b}\right]}=k_{s}\frac{{\left(1/\left[m_{b}\right]\right)}^{n}}{{\left(K_{mb}\right)}^{n}+{\left(1/\left[m_{b}\right]\right)}^{n}}+k_{min}^{s}, \end{array}$$ (23)

where *k*_*s*_ and $k_{min}^{s}$ are positive constants, 1/*K*_*mb*_ is the critical threshold concentration and *n* is the Hill coefficient.

In summary, the concentration of actin filaments is determined by averaging the fluid pressure distribution along the cell membrane. In case of high sensing pressure near normal cells, the kinetics of actin and myosin allows the cell to accumulate the bound myosin II, which lowers the stiffness of the nucleus. This low stiffness results in lower elastic force of the nucleus and thus the nucleus deforms easily when it passes through the gap between normal cells. The deformation of nucleus affects the surrounding fluid vice versa.

**Fig 5:**
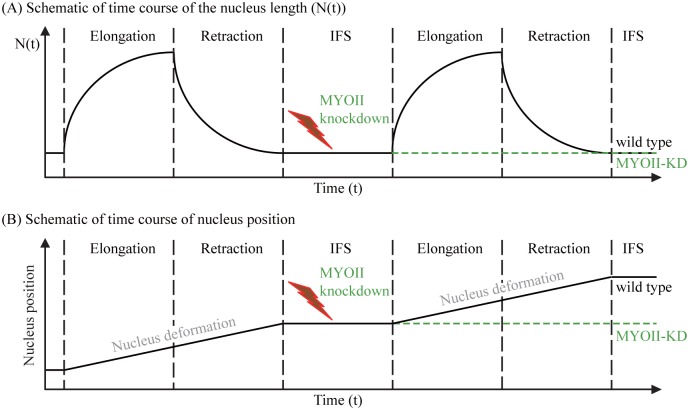
Schematics of the deformation of nucleus in wild type and MYOII-KD. Schematic of changes in the nucleus length (*N*(*t*) in (A)) and nucleus position (B) for the wild type and MYOII-KD case. IFS stands for the intermediate free space.

Fig 5 shows schematics of myosin II-mediated changes in the nucleus length (Fig 5A) and nucleus position (Fig 5B) for the wild type and myosin II knockdown (MYOII-KD) cases. For the wild type, the nucleus length *N*(*t*) shows same patterns as the cell body length in Fig 4A over a cycle of elongation (increase) and retraction (decrease) steps during its migration through a narrow gap. The nucleus position is a monotone increasing function of time during this cycle (Fig 5B). When the cell migrates through the intermediate free space (IFS) without physical barriers, the nucleus deforms into the reference configuration. During this IFS period, both the nucleus length and position remain to be constant, waiting for the next cycle. The same spring-like pattern in wild type resumes again in the presence of the physical barrier. When the myosin II is suppressed by an inhibitor such as blebbistatin (red thunder), both the nucleus length (green dotted line in Fig 5A) and its position (green dotted line in Fig 5B) remain steadfast for the rest of cycles.

### Reaction-diffusion of chemoattractants

Multiple biophysical and biochemical signals affect glioma cell migration in the brain [4]. These include haptotactic process [2, 46, 47] in the brain, the EGF family [3], indirect signaling through other cell types [5], the TGF-*β* family [48], PDGF [11], scatter factor/hepatocyte growth factor (SF/HGF) [49], SDF-1 [50], and certain lipids [51]. For simplicity, we assume here that (i) there is only one chemotactic source at a fixed location, (ii) a glioma cell responds to the chemotactic signal and initiates the migration toward the up-gradient of the chemoattractant (chemotaxis) by using myosin II dynamics, (iii) there is no flux of the chemoattractant at the outer boundary (∂*Ω*) of the computational domain. These assumptions lead to the following equations with the initial and no flux boundary conditions for the concentration *C*(***x***, *t*) of chemoattractants in space ***x*** and time *t*,

$$\begin{array}{c} \frac{\partial C}{\partial t}=\underset{\text{Diffusion}}{\underset{︸}{D_{C}\Delta C}}+\underset{\text{Injection}}{\underset{︸}{\lambda_{in}^{C}I_{\Omega_{\epsilon }}}}-\underset{\text{Decay}}{\underset{︸}{\mu_{C}C}}\;\text{in}\;\Omega , \end{array}$$ (24)

where *D*_*C*_ is the diffusion coefficient of chemoattractants, $\lambda_{in}^{C}$ represents the strength of the chemotactic source, *I*_*Ω*_*ϵ*__(⋅), is an indicator function on the source *Ω*_*ϵ*_, *μ*_*C*_ is the decay rate of the chemoattractant. We assume that the active force constant *c*_*a*_ in Eq (11) is proportional to the chemical gradient |***F***_*C*_| as follows:

$$\begin{array}{c} c_{a}=\chi (\alpha +|F_{C}\left|\right), \end{array}$$ (25)

where *χ* is the basal active force strength, *α* is the basic signaling strength in the microenvironment and is chosen to be 0.15. Here ***F***_*C*_ is given by

$$\begin{array}{c} F_{C}=\frac{\nabla C}{\sqrt{1+\lambda_{s}{\left|\nabla C\right|}^{2}}},\;\;\lambda_{s}\in R^{+}, \end{array}$$ (26)

where *λ*_*s*_ is the chemotactic parameter set equal to 20, for simplicity. Here, |***F***_*C*_| represents the strength of the chemical gradient and remains bounded, even though |∇*C*| may become very large [14].

The parameter values and the reference values for Eqs (1)–(26) are given in Table 1. Some of the parameter values are taken from the literature and the rest are estimated in this work.

## Results

### Dynamics of the actin-myosin machinery

**Fig 6:**
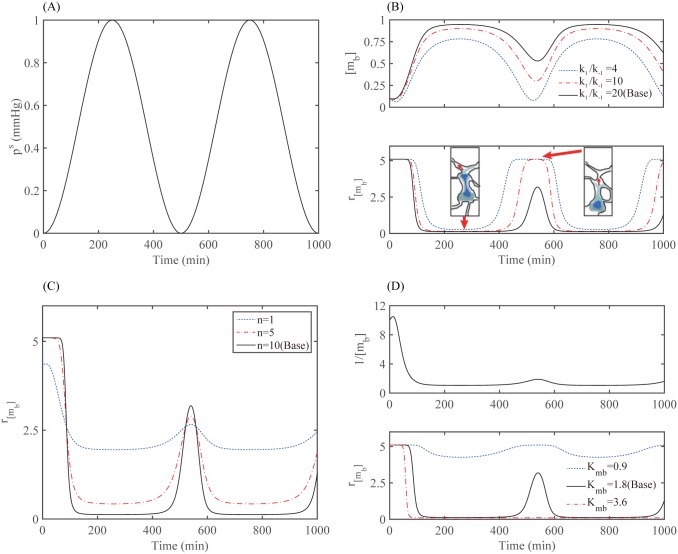
Dynamics of the acto-myosin module in response to fluctuating sensing pressure in the microenvironment. (A) Periodic sensing pressure averaged over the cell membrane. (B) Time courses of myosin II concentrations ([*m*_*b*_]; upper panel) and stiffening rates of the nucleus (*r*_[*m*_*b*_]_; lower panel) for various ratios of *K* (*K* = *k*_1_/*k*_−1_ = 4, 10, 20) in response to the fluctuating sensing pressure *p*^*s*^(*t*) in (A). (C) Time courses of stiffening rates of the nucleus for various Hill coefficient (*n* = 1, 5, 10) in response to the fluctuating sensing pressure *p*^*s*^(*t*) in (A). (D) Time courses of 1/[*m*_*b*_] (upper panel) and stiffening rates of the nucleus (*r*_[*m*_*b*_]_; lower panel) for various values of *K*_*mb*_ (*K*_*mb*_ = 0.9, 1.8, 3.6) in response to the fluctuating sensing pressure *p*^*s*^(*t*) in (A).

In Fig 6 we analyze the acto-myosin dynamics. The steady state of the bound myosin concentration ([*m*_*b*_]) in the Eq (18) is given by

$$\begin{array}{c} {\left[m_{b}\right]}^{s}=\frac{k_{1}\left[m_{T}\right]\left[a\right]}{k_{1}\left[a\right]+k_{-1}}=\left[m_{T}\right]\frac{\left[a\right]}{[a]+1/K} \end{array}$$ (27)

where $K=\frac{k_{1}}{k_{-1}}$ is the ratio of the association rate (*k*_1_) to the dissociation rate (*k*_−1_). In our model, the concentration of actin filament is linearly proportional to the sensing pressure from the microenvironment, especially from the narrow intercellular gaps between normal brain cells. In response to the periodic change in sensing pressure (*p*^*s*^; Fig 6A) averaged over the cell membrane, the concentration of bound myosin II ([*m*_*b*_]) is increased or decreased (Fig 6B) for various *K* values ($K=\frac{k_{1}}{k_{-1}}=4,10,20$). In our model, since the stiffening rate of the nucleus and the bound myosin II are out of phase, the up-regulated expression of the bound myosin II on time intervals [150 *min*, 450 *min*] and [650 *min*, 850 *min*] induces low stiffening rate of the nucleus (*r*_[*m*_*b*_]_), resulting in the deformation of the nucleus (see the first sub-figure in the bottom panel in Fig 6B). On the other hand, the down-regulated expression level of the myosin II near *t* = 0, 500, 1000 *min* recovers close to the original stiffening rate of the nucleus (*r*_[*m*_*b*_]_), resulting in the rigid non-deformed nucleus (see the second sub-figure in the bottom panel in Fig 6B). In Fig 6C, we investigated the mechanical response (stiffening rate *r*_[*m*_*b*_]_) of the nucleus for various Hill coefficients *n* (*n* = 1, 5, 10). Lowering the coefficient *n* leads to smaller changes in the stiffening rate. In particular, the nucleus will not deform much for the low *n* value (*n* = 1; blue dotted curve) even in the presence of the high sensing pressure (*p*^*s*^). Fig 6D shows time courses of 1/[*m*_*b*_] (upper panel) and stiffening rates of the nucleus (*r*_[*m*_*b*_]_; lower panel) for various values of *K*_*mb*_ (*K*_*mb*_ = 0.9, 1.8, 3.6) in response to the fluctuating sensing pressure *p*^*s*^(*t*). The stiffening rate of the nucleus (lower panel in Fig 6D) changes dramatically in response to the same signal from the bound myosin II ([*m*_*b*_]) (upper panel in Fig 6D) for different values of *K*_*mb*_. The nucleus does not mechanically respond to the signal for the smaller value (*K*_*mb*_ = 0.9; blue dotted curve) with the persistent high stiffening rate. On the other hand, the nucleus becomes too soft without returning to the basic stiff state even in the absence of physical barrier for a larger value (*K*_*mb*_ = 3.6; red dash-dotted curve). With the base value (*K*_*mb*_ = 1.8; black solid curve), the nucleus deformation is coordinated dynamically in response to the fluctuating sensing pressure (*p*^*s*^) from the microenvironment. This mechanical adaptation is a necessary condition for cell infiltration through the narrow intercellular space [11] (see Fig 2).

### Glioma cell migration through a dense network of normal brain cells

**Fig 7:**
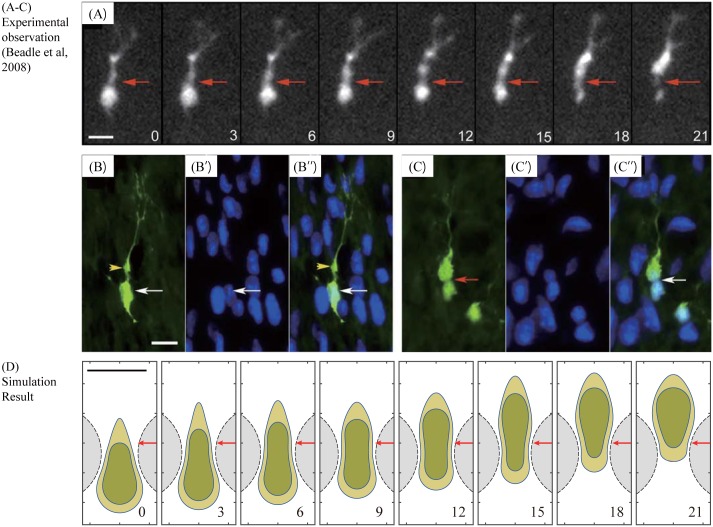
Experiments and simulation results using a mathematical model. (A-C) Experimental results of a GFP-expressing rat glioma cell. Time courses of the profiles of a moving glioma cell in (A) show the deformation of the cell body during cell translocation. Micrographs taken at 3-min intervals (*t* = 0, 3, 6, 9, 12, 15, 18, 21 *min*). Red arrowhead = a focal point between the cell body and the swelling in the leading edge. Micrographs of a section stained for GFP (green; moving glioma cell) and DAPI (blue; resident brain cells) show GFP-expressing glioma cells at distinct two phases of the migration in (B,C). While the nucleus (white arrow) is separated from a prominent dilatation at the front (yellow arrowhead) in the first step (*B* − *B*′′), a focal deformation of the nucleus (red arrow) and cell body is observed in the next step (*C* − *C*′′). Bars, 10*μm*. Reprinted from Beadle C, Assanah M, Monzo P, Vallee R, Rosenfield S, et al. (2008) The role of myosin II in glioma invasion of the brain. Mol Biol Cell 19: 3357-3368 [11] under a CC BY license, with permission from American Society for Cell Biology, original copyright 2008. (D) Time courses of cell morphology as a glioma cell migrates through a narrow gap between two glial cells in the brain. Bars, 10*μm*. Profiles of a glioma cell with deforming nucleus (dark green) at *t* = 0, 3, 6, 9, 12, 15, 18, 21 *min* are shown in the presence of two normal cells (gray region with dotted line cell boundaries).

Beadle *et al.* [11] showed the experimental observation on the cell migration of a GFP-expressing rat glioma cell through a dense network of normal cells in the rat brain in Fig 7A–7C. The cell kymograph in Fig 7A depicts several features that occur during this invasion process by a DsRed-labeled glioma cell through the brain tissue. A leading cytoplasmic component extends forward and begins to develop a proximal dilatation, which is similar to ones observed in neural progenitor cell migration in early postnatal brain [10, 52–54]. This cytoplasmic deformation illustrates dynamic processes of retraction, extension, and branching as the moving cell defines the migration pathway [11]. This is naturally followed by forward movement of the cell body via dumbbell shape or ‘hourglass’ deformation (Fig 7A). Staining of the thin sections of fixed tissue in the experiments [11] also illustrates the same hourglass deformation of the cell nucleus at the invasive margin where the moving glioma cell is trapped by normal brain cells and their densely packed cytoplasmic parts (Fig 7B–7B′′ and 7C–7C′′). In Fig 7D we show simulation results on cell morphology at *t* = 0, 3, 6, 9, 12, 15, 18, 21 *min* as a glioma cell migrate through a narrow gap between two glial cells in the brain. It illustrates how a glioma cell biomechanically deforms its cell body (light yellow) and nucleus (dark green) using the acto-myosin machinery in order to infiltrate the narrow space between two normal cells (gray region with dotted line cell boundaries). This mechanical response is in good agreement with experimental observations in Fig 7A by Beadle *et al.* [11].

**Fig 8:**
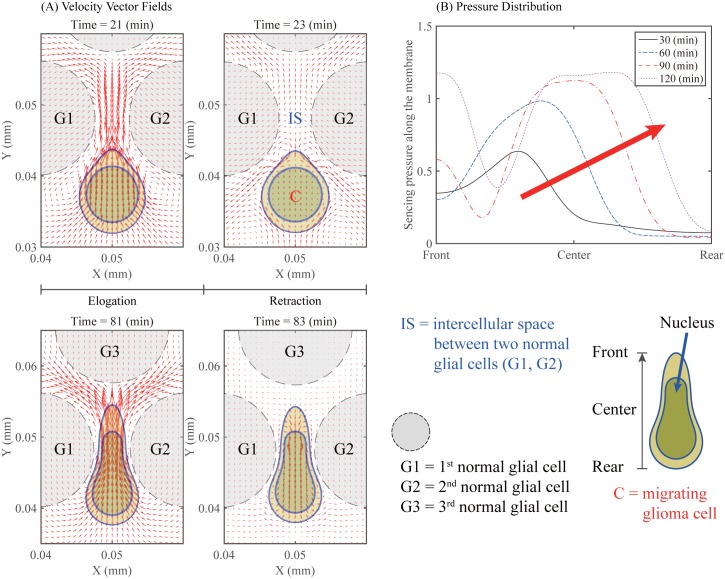
Dynamics of elongation and retraction of a infiltrating glioma cell in a narrow intercellular gap (IS) between normal glial cells. (A) Cell deformation and velocity field (red arrows) near a moving glioma cell (double blue solid curves) and normal glial cells (dashed circles) during the elongation steps at *t* = 21, 81 *min* and retraction steps at *t* = 23, 83 *min*. (B) Spatial distribution of pressure along the cell membrane at *t* = 30, 60, 90, 120 *min* as it pushes through a narrow gap.

Fig 8A shows the dynamics of elongation and retraction steps of a glioma cell (marked in ‘C’) migrating through an intercellular space (IS) between normal glial cells (marked in ‘G1’ and ‘G2’). The velocity field (red arrows) near the infiltrating glioma cell (double blue solid curves) and normal glial cells (dashed circles) indicates the attachment of the rear and strong protrusion of the front during the elongation steps at *t* = 21, 81 *min*. On the other hand, during the retraction steps at *t* = 23, 83 *min*, the vector field shows the weak inward flow at the front due to adhesion of the front and outward flow at the back by releasing the rear. Fig 8B shows the spatial distribution of pressure along the cell membrane from the front to the rear, as it pushes through a narrow gap at *t* = 30, 60, 90, 120 *min*. The relatively low pressure distribution at *t* = 30 *min* illustrates the initial build up of pressure at the front of the cell body near the entrance as the cell enters the narrow gap between two glial cells. As the glioma cell migrates through the intercellular space, the peak of pressure travels toward the rear part of the cell body, generating a traveling wave of pressure along the cell body due to the physical barrier. This traveling wave indirectly reflects the mechanical pressure the moving cell feels as it passes through the narrow gap. This type of glioma cell deformation from active migration [11] is tightly regulated by acto-myosin machinery and is qualitatively different from passive cell deformation due to physical forces resulting from external stresses [55].

### Effect of myosin II on deformation of nucleus for cell infiltration

**Fig 9:**
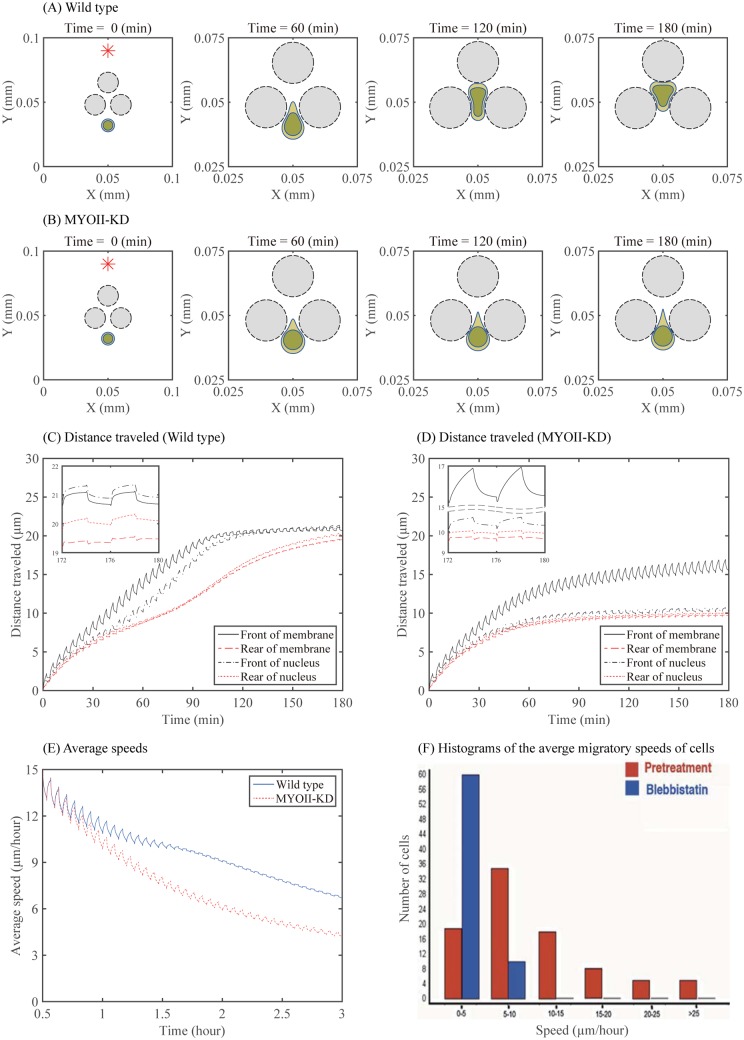
Dynamics of cell infiltration in wild type and MYOII-KD case. (A,B) Time evolution of profiles of migratory glioma cells (blue circles) in the presence (wild type in (A)) and absence (MYOII-KD in (B)) of myosin II at *t* = 0, 60, 120, 180 *min*. Black circle = the normal glial cells, blue outer circle = the membrane of a glioma cell, blue inner circle = nucleus of a glioma cell. (C,D) Time courses of the distance traveled (the front and rear of the cell membrane and nucleus) in wild type (C) and MYOII-KD (D). (E) Average speed of the glioma cell for the wild type and MYOII-KD. (F) Histograms of the average migratory speeds of rat glioma cells in the absence (red) and presence of (blue) 50 *μM* blebbistatin, a biochemical inhibitor of myosin II. The average cell speed is decreased in the presence of the inhibitor, blebbistatin. Reprinted from Beadle C, Assanah M, Monzo P, Vallee R, Rosenfield S, et al. (2008) The role of myosin II in glioma invasion of the brain. Mol Biol Cell 19: 3357-3368 [11] under a CC BY license, with permission from American Society for Cell Biology, original copyright 2008.

**Fig 10:**
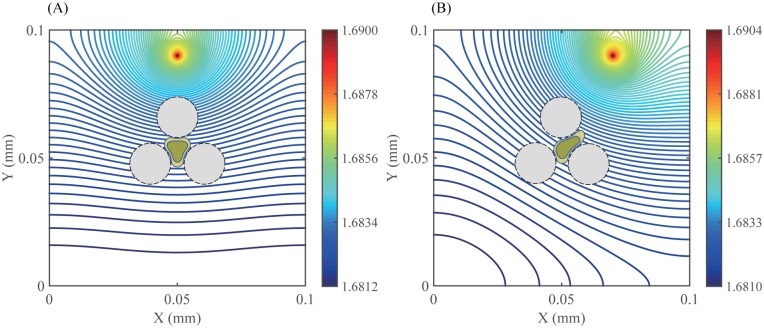
Spatial dynamics of chemoattractant. (A,B) Profiles of chemoattractants at *t* = 180 *min* when the source of the chemoattractant was located (A) in the middle of top, where (*x*, *y*) = (0.05, 0.09) and (B) near the top right of the domain, where (*x*, *y*) = (0.07, 0.09).

To investigate the role of myosin II, we compare the dynamics of a glioma cell in wild type and myosin II knockdown (MYOII-KD) by setting the myosin-pressure sensitivity equal to *k*_*p*_ = 0.9 for wild type and *k*_*p*_ = 0.045 for MYOII-KD given in Eq (21). Fig 9A and 9B show the movement of a migratory glioma cell (blue circles) through the normal glial cells (black dashed circles) in the brain tissue in cases of wild type (Fig 9A) and MYOII-KD (Fig 9B) at four different times *t* = 0, 60, 120, 180 *min*. The glioma cell is attracted to the chemoattractant (red star), located at the top of the domain, that diffuses through the brain tissue. Specifically the chemotactic point source was placed at (0.05, 0.09) ∈ *Ω* = [0, 0.1] × [0, 0.1](*mm* × *mm*). See Fig 10A for the spatial profile of chemoattractants at *t* = 180 *min*. Whereas the glioma cell is not able to migrate between two normal brain cells in the MYOII-KD case, the large deformation of the nucleus due to myosin II enables the cell to squeeze through the normal cells in the wild type. Fig 9C and 9D show the traveled distance of the front and back of the cell membrane and nucleus in the wild type and MYOII-KD, respectively. Fig 9E shows the average speed of the glioma cell for the wild type and MYOII-KD. It is evident that the wild type glioma cell is more efficient in motility than the MYOII-KD cell type. It is known that cell speeds depend on many factors such as growth factors and cell culture conditions. Various cell speeds of glioma cells were reported in literature [15] and therein. Cell speeds in glioma have been experimentally measured to be in the range of 15-20 *μm*/*h* in 3D glioblastoma culture with/without EGF-stimulation and 39-45 *μm*/*h* in 2D barrrier-free culture [56], 15-25 *μm*/*h* in glioblastoma cells in the absence/presence of *α*-actinin isoforms [57], and 15-48 *μm*/*h* in collagen I matrix [58]. In our simulations, the cell speed in wild type ranges from 7-15 *μm*/*h* which lies in the lower range of the experimental speed due to the surrounding obstacles in the environment (Fig 9E). Fig 9F shows the average migratory speeds of rat glioma cells in the absence (red) and presence (blue) of 50 *μM* blebbistatin, a biochemical inhibitor of myosin II, in the experiments by Beadle *et al.* [11]. One can see that the peak of the cell distribution against the average cell speed in the wild type is shifted to the lower range of 0-5 *μm*/*h* in the MYOII-KD case. In our simulations (Fig 9E), the average speed in the MYOII-KD case decreases over time and reaches in the range of 0-5 *μm*/*h* at *t* = 3 *h*, in which the average speed of the wild type reaches in the range of 5-10 *μm*/*h* at *t* = 3 *h*. This decreased cell speed in the presence of the inhibitor is in good agreement with experimental data by Beadle *et al.* [11] (Fig 9F).

**Fig 11:**
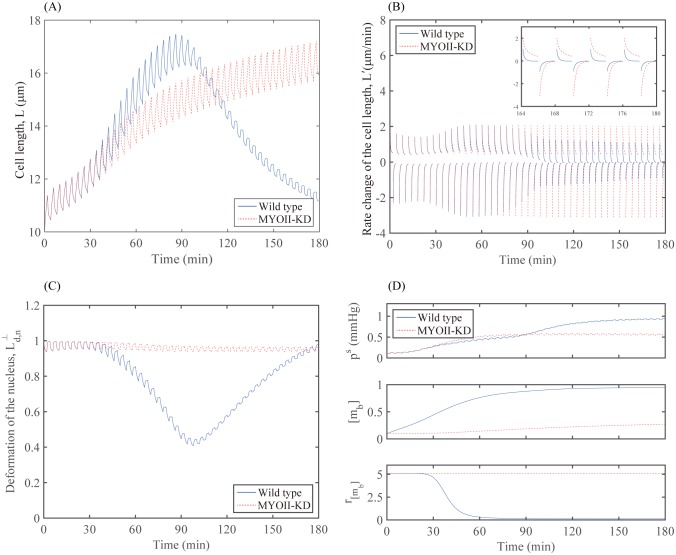
Bio-mechanical responses in wild type and MYOII-KD case. (A,B) Time courses of the cell length *L* (A) and the rate change *L*′(*t*) of the cell length (B) in wild type and MYOII-KD. (C) Time courses of deformation $L_{d,n}^{⊥}$ of the nucleus in wild type and MYOII-KD. (D) Time courses of the average sensing pressure (*p*^*s*^; top panel), myosin II level ([*m*_*b*_]; middle panel), and the stiffening rate (*r*_[*m*_*b*_]_; bottom panel) in wild type and MYOII-KD.

In Fig 11A and 11B, we show time courses of the cell length (*L*) in the migration direction and its rate change (*L*′), corresponding to the wild type and MYOII-KD glioma cells simulated in Fig 9. During the elongation phase, the rate *L*′ starts from the maximum positive value and decreases to zero (see the enlarged view in Fig 11B). As the cell switches its migration step to the retraction phase, the rate *L*′ starts from the minimum negative value and increases to zero (see the enlarged view in Fig 11B). The glioma cell alternates these two phases repeatedly and creates cycles. Local peaks and troughs in Fig 11A correspond to these rate changes throughout cell migration. In the case of the wild type, the cell body length (*L*) slowly increases overall during the first half of the infiltration step and reaches its maximum value around *t* = 90 *min* because of the elongation and deformation of its cell body and nucleus due to the narrow intercellular gap between two normal cells (Fig 9A). For the second half of the infiltration step, the cell body length (*L*) slowly returns back to the normal range around *t* = 180 *min* because of the successful passage of the gap. However, the overall cell body length (*L*) increases and levels off in the MYOII-KD case because of the unsuccessful infiltration of the glioma cell under the physical constraints. See Fig 9B.

Fig 11C shows time courses of the averaged deformation rate $L_{d,n}^{⊥}$ of the nucleus in the direction ${\vec{d}}^{⊥}$ that is perpendicular to the migration direction $\vec{d}$. Here we define the averaged deformation rate relative to the initially designated distance by,

$$\begin{array}{c} L_{d,n}^{⊥}\left(t\right)=\frac{1}{2}\left(L_{d,n}^{⊥,W}\left(t\right)+L_{d,n}^{⊥,E}\left(t\right)\right), \end{array}$$ (28)

$$\begin{array}{c} L_{d,n}^{⊥,W}\left(t\right)=\frac{|X_{n}^{W}\left(t\right)-X_{nc}\left(t\right)|}{|X_{n}^{W}\left(0\right)-X_{nc}\left(0\right)|},\;\;L_{d,n}^{⊥,E}\left(t\right)=\frac{|X_{n}^{E}\left(t\right)-X_{nc}\left(t\right)|}{|X_{n}^{E}\left(0\right)-X_{nc}\left(0\right)|}, \end{array}$$ (29)

where ***X***_*nc*_(*t*) is a trajectory of a marker point initially located in the center of the nucleus and $X_{n}^{W}\left(t\right)$ and $X_{n}^{E}\left(t\right)$ are trajectories of two boundary points initially located at the farthest ends of the nucleus in the direction of west and east, respectively. This implies that, if we assume that the cell deforms symmetrically in time, the cell deforms outward if $L_{d,n}^{⊥}\left(t\right)>1$, the cell deforms inward if $L_{d,n}^{⊥}\left(t\right)<1$, and the cell stays the same if $L_{d,n}^{⊥}\left(t\right)=1$. In our simulations, the inward deformation of the cell is observed at all times, as the cell moves and passes through the narrow intercellular gap between normal cells. As shown in Fig 11C, the cell in the wild-type deforms inward with a large degree, whereas the cell in MYOII-KD does not deform much. Fig 11D shows the sensing pressure (top panel), myosin II concentration (middle panel), and the stiffening rate (bottom panel) for wild type and MYOII-KD. Temporal changes in sensing pressure lead to different patterns of myosin II levels. In wild type, the myosin II level increases significantly, whereas the myosin II level in MYOII-KD decreases and remains at a lower level. As a result, the stiffening rate of nucleus in wild type dramatically drops down to a lower level as [*m*_*b*_] increases. However, in MYOII-KD it maintains a high level of the stiffening rate for all times. The mechanical constraints induced from the narrow intercellular spaces between two normal cells interrupt the movement of the cell body due to the undeformed nucleus when the myosin II was knocked down. Therefore, the cell migration is blocked near the entrance to the extracellular space between the gap, even though the cell senses the mechanical pressure in the migrating front. In the case of the wild type, the acto-myosin machinery is activated in response to the mechanical pressure (top panel; Fig 11D) at the front of the migratory cell and the up-regulated myosin II level (middle panel; Fig 11D) provides the necessary force to squeeze and deform the nucleus through the narrow extracellular space between two cells.

**Fig 12:**
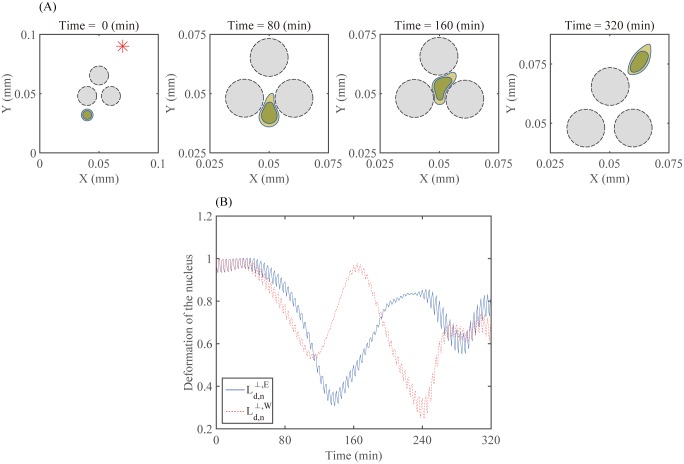
Cell infiltration in response to the chemoattractant. (A) Time evolution of profiles of a migratory glioma cell (blue circles) at *t* = 0, 80, 160, 320 *min* in response to the gradient of a chemoattractant on the top-right corner of the domain (red star). Black circle = the normal glial cells, blue outer circle = the membrane of a glioma cell, blue inner circle = nucleus of the glioma cell. (B) Time courses of the deformation of the nucleus to the left ($L_{d,n}^{⊥,W}$) and to the right ($L_{d,n}^{⊥,E}\left(t\right)$).

Fig 12A shows the motion of a migratory glioma cell (wild type) at *t* = 0, 80, 160, and 320 *min* in response to the chemotactic source (red star) located at the top-right corner of the computational domain. After successfully passing the narrow extracellular region, the cell is infiltrating another narrow extracellular region in response to the strong chemical signal from the top-right. See Fig 10B for the spatial profile of chemoattractants at *t* = 180 *min*. Fig 12B shows the deformation rate of the nucleus with respect to the left ($L_{d,n}^{⊥,W}$) and right ($L_{d,n}^{⊥,E}$) relative to the migration direction. The patterns of $L_{d,n}^{⊥,W}$ and $L_{d,n}^{⊥,E}$ are initially the same and then evolve to very different patterns as the cell decides to migrate to the right and break the symmetry of the migration direction. This predicts that the cell is able to quickly use the acto-myosin machinery in order to deform both its cell body and nucleus effectively and redirect its movement according to the positive chemoattractant gradient in this complex and harsh microenvironment.

### Cell migration through multiple layers of cells

**Fig 13:**
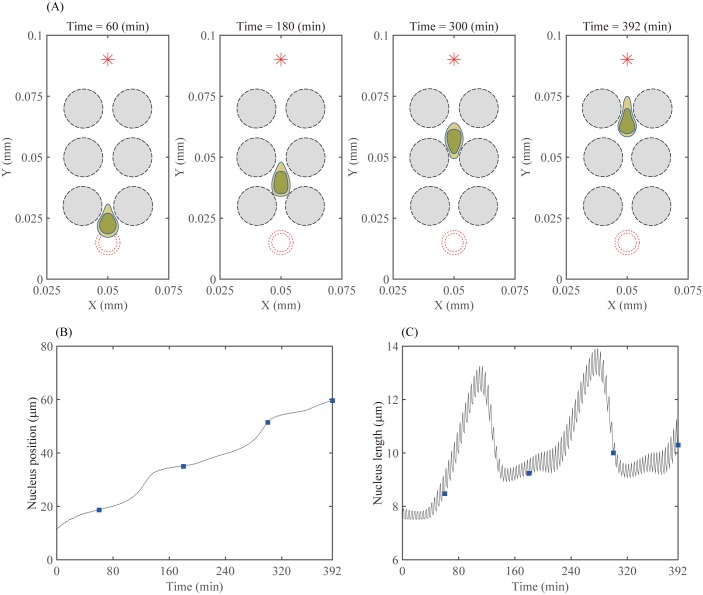
Dynamics of cell infiltration in multiple layers of normal cells. (A) Time evolution of profiles of a migratory glioma cell (blue circle) through a network of normal cells (black circles) at times *t* = 60, 180, 300, 392 *min*. Red star (*) = a chemotactic source, red circles = initial configuration of the glioma cell. (B,C) Time courses of the nucleus position and the longitudinal length of the nucleus of the glioma cell, respectively. Filled squares indicate the times given in (A). The nucleus position is obtained from keeping track of the *y*-component of the bottom point of the nucleus and the longitudinal nucleus length is measured by the distance between the top and bottom points of the nucleus.

In Fig 13A we show a typical pattern of a moving glioma cell through a network of normal cells in the brain. The mechanical constraints generated from the narrow intercellular spaces between normal cells inhibit the forward movement of the cell body and nucleus until the up-regulated myosin II level provides the necessary force to squeeze the nucleus through the narrow space. The relatively stationary nucleus position observed as the cell moves through the narrow intercellular space is followed by a rapid upward movement as the cell moves through the intermediate free space, whereas the overall nucleus length steadily increases during elongation step and then decreases during retraction step, resulting in saltatory movement [11], see Fig 13B and 13C.

Note, however, that glioma cells crawling on a two-dimensional solid substrate without spatial obstacles have been shown to move in a different manner, similar to cell migration of fibroblasts, in which case cells make use of a broad lamellipodium and thus the nucleus remains undistorted, which results in a continuous, uninterrupted, forward movement [11]. Therefore, this movement is characterized by the linear increase of nuclear position over time, which demonstrates that myosin II plays a passive role in maintaining the cell polarity and shape, but its activity is not necessary for cell motility in this friendly, barrier-free, microenvironment.

### Infiltration in challenging microenvironment and predictions of the model for an anti-invasion therapy

As mentioned in Introduction Section, the regrowth of dispersed individual glioma cells is blamed for the low survival rate in patients with glioblastoma. Anti-invasion therapies targeting ECM, integrins, proteases, signaling networks, the cytoskeleton, and ion channels [11, 59–65] or localization of dispersed glioma cells [15, 16, 18, 19] may be considered as possible therapeutic strategies to eradicate invisible glioma cells.

**Fig 14:**
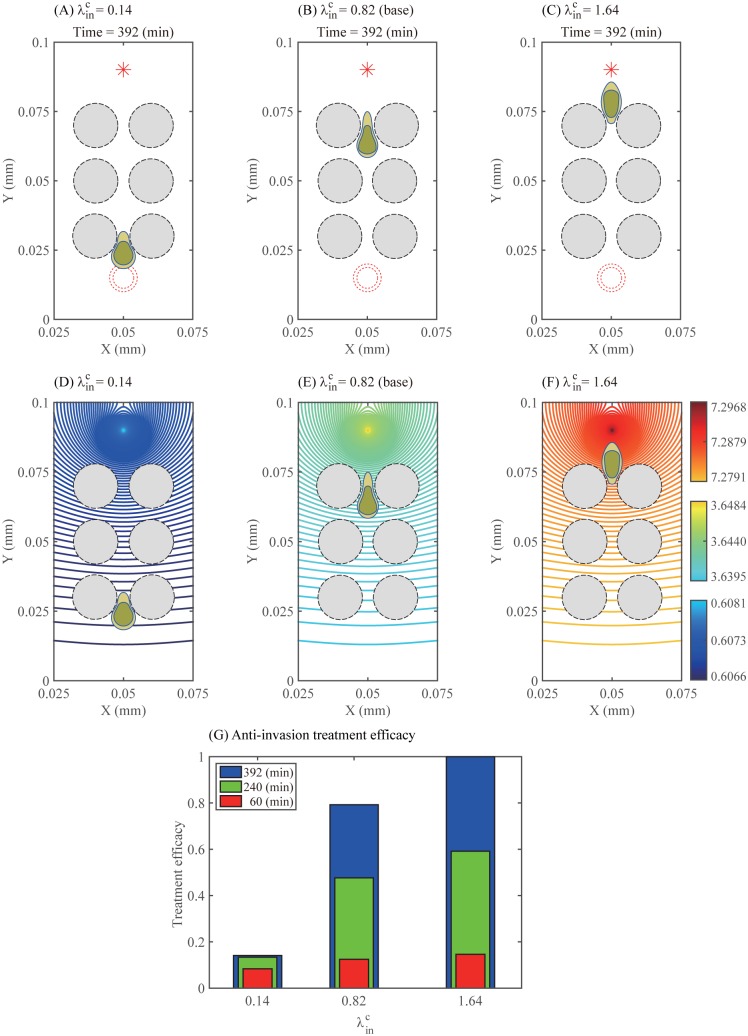
Therapeutic strategies (Localization of the glioma cells). (A-C) Effect of the different strength $\lambda_{in}^{C}$ of the chemotactic source on migration patterns of a glioma cell (wild type; blue curves) at the final time *t* = 392 *min*: $\lambda_{in}^{C}$ = 0.14 (A), 0.82 (B), 1.64 (C). Red star (*) = a chemotactic source, red circles = the initial configuration of a glioma cell. (D-F) Profiles of the chemoattractant at the final time for the corresponding three cases in (A-C). (G) Anti-invasion treatment efficacy for those three cases in (A-C).

**Fig 15:**
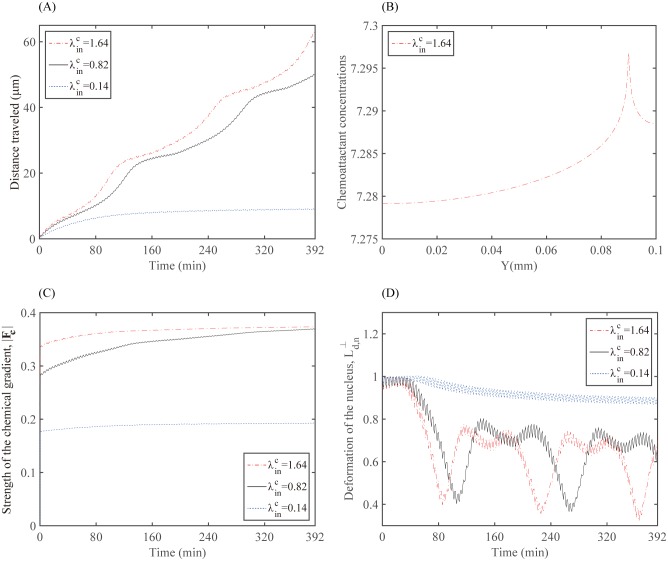
Dynamics for various strengths of chemotactic sources. (A) Migration distance for various strengths of chemotactic sources ($\lambda_{in}^{C}$ = 0.14, 0.82, 1.64). (B) Concentration level of the chemoattractant along the vertical center line at *t* = 392 *min* when $\lambda_{in}^{C}$ = 1.64. (C) Time courses of chemotaxis-driven active force strength (|***F***_*C*_|). (D) Time courses of deformation $L_{d,n}^{⊥}$ of the nucleus.

**Fig 16:**
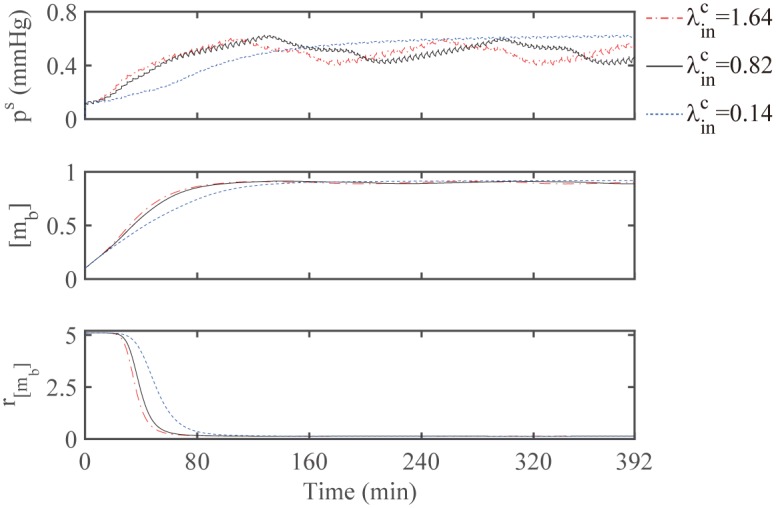
Internal acth-myosin dynamics for various strengths of chemotactic sources. Time courses of the average sensing pressure (*p*^*s*^; top panel), the concentration of bound myosin II ([*m*_*b*_]; middle panel), and the stiffening rate (*r*_[*m*_*b*_]_; bottom panel) during the cell migration for three cases in Fig 14.

As a potential therapeutic strategy, our simulations demonstrate that the speed of a localization process of scattered glioma cells can be expedited as the chemotactic signal is strengthened. Fig 14A–14C compare the migration speed of a glioma cell through a dense network of normal cells in the brain at the final time *t* = 392 *min* in response to low ($\lambda_{in}^{C}=0.14$; Fig 14A), normal ($\lambda_{in}^{C}=0.82$ (base); Fig 14B), and high ($\lambda_{in}^{C}=1.64$; Fig 14C) strengths of chemotactic sources inoculated at the middle top of the computational domain. Fig 14D–14F show the corresponding profiles of the chemoattractant concentration at the final time *t* = 392 *min* for those three cases. As the source strength ($\lambda_{in}^{C}$) is increased, the chemoattractant level at the cell site is increased and the chemoattractants diffuse rapidly, and the glioma cell responds promptly and moves longer distance ($\lambda_{in}^{C}=1.64$; Fig 14C). However, the cell motility is inhibited when the chemotactic signal is relatively weak ($\lambda_{in}^{C}=0.14$, Fig 14A). Anti-invasion treatment efficacy is shown in Fig 14G. This implies that the best result in terms of the localization of tumor cells would come out when the chemoattractant is injected on the periphery of the resection site at the highest rate ($\lambda_{in}^{C}=1.64$). Fig 15A displays time courses of the distance traveled for the above three cases and Fig 15B shows the concentration level of chemoattractants along the vertical center line when $\lambda_{in}^{C}=1.64$. The chemoattractant source generates a gradient of the chemoattractant for the cell to migrate, while the larger source strength induces the higher concentration level. Fig 15C shows time courses of the chemotaxis-driven active force strength (|***F***_*C*_|) from the chemotactic gradient (∇*C*) at the cell front. As the chemotactic strength $\lambda_{in}^{C}$ is increased, |***F***_*C*_| is increased, leading to longer distance traveled by the cell (Fig 15A). Fig 15D shows time courses of the nucleus deformation $L_{d,n}^{⊥}$. Cyclic events of large inward deformation of the nucleus are observed whenever the cell passes through the intercellular gap between two normal cells during cell migration. For example, see three troughs at different time points (*t* = 86, 226, 366 *min*) when $\lambda_{in}^{C}=1.64$ (red dash-dotted line). This cyclic phenomenon appears when the average sensing pressure, subsequently the concentration of bound myosin II, and the chemotaxis-driven active force fluctuate in the course of cell migration, see Fig 16. Notice the lowered stiffening rate near the corresponding times are observed when $\lambda_{in}^{C}=1.64$, which corresponds to the duration for the shape deformation of the cell body and nucleus in Fig 14C.

**Fig 17:**
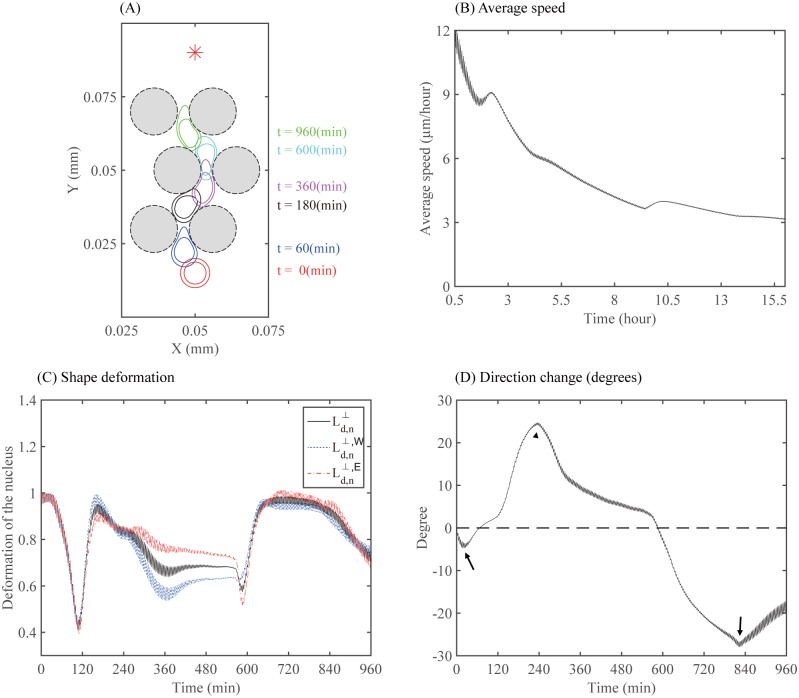
Cell migration through complex microenvironment. Migration of a tumor cell through a dense network of normal cells in the brain. The normal cells were placed in a zig-zag fashion and the chemotactic source was located at the top of the computational domain. (A) A profile of the migratory glioma cell through normal cells (black dashed circles) at *t* = 0 (red solid), 60 (blue solid), 180 (black solid), 360 (cyan solid), 600 (light blue solid), 960 (green solid) *min*. (B) Time courses of the averaged speed. (C) Time courses of shape deformations ($L_{d,n}^{⊥},L_{d,n}^{⊥,W},L_{d,n}^{⊥,E}$) of the nucleus. (D) Direction change (angle from the vertical axis) of the cell shown in panel (A) over the time course of 960 *min*. Positive values indicate that the cell moves in the north-east direction and negative values indicate that the cell moves in the north-west direction. The black arrows and arrowheads in (D) mark the corresponding times at which the cell changes its moving direction.

Glioma cells can also effectively adapt to the harsh microenvironment. In Fig 17A we show a time profile of a glioma cell that is infiltrating into the brain tissue by squeezing through the narrow extracellular path between normal cells (black dashed circles). The harsh microenvironment is represented as spatial constraints of normal cells prescribed in a zig-zag fashion. Solid circles in different colors indicate the deformed membrane and nucleus of the cell at *t* = 0 (red solid), 60 (blue solid), 180 (black solid), 360 (magenta solid), 600 (cyan solid), 960 (green solid) *min*. The cell changes its migration direction effectively based on chemotactic signals that diffuse from the top of the domain. The shape deformation of the cell body and nucleus is due to the effective use of myosin II whenever the cell feels the mechanical pressure in the intercellular path, reflecting the amoeboid motility [11]. Fig 17B illustrates the averaged speed of the cell. The cell spends much time and energy whenever it faces the obstacles (narrow gaps between normal cells) and needs to deform the cell membrane and nucleus for the infiltration process. This leads to the relatively low speed of the cell. Once the cell is freed from the narrow path, it can easily move forward, which induces the higher cell speed. The presence of normal cells distributed on regular spatial intervals induces the cycle of the fast and slow cell migration pattern while the averaged cell speed is in good agreement with experimental data [9]. This periodic deformation of the cell body and nucleus is also shown in Fig 17C. Fig 17D illustrates the measurement of angle from the vertical axis to show the change in the migration direction of the cell shown in Fig 17A over the time course of 960 *min*. The black arrows and arrowheads mark the corresponding times at which the cell changes its moving direction.

**Fig 18:**
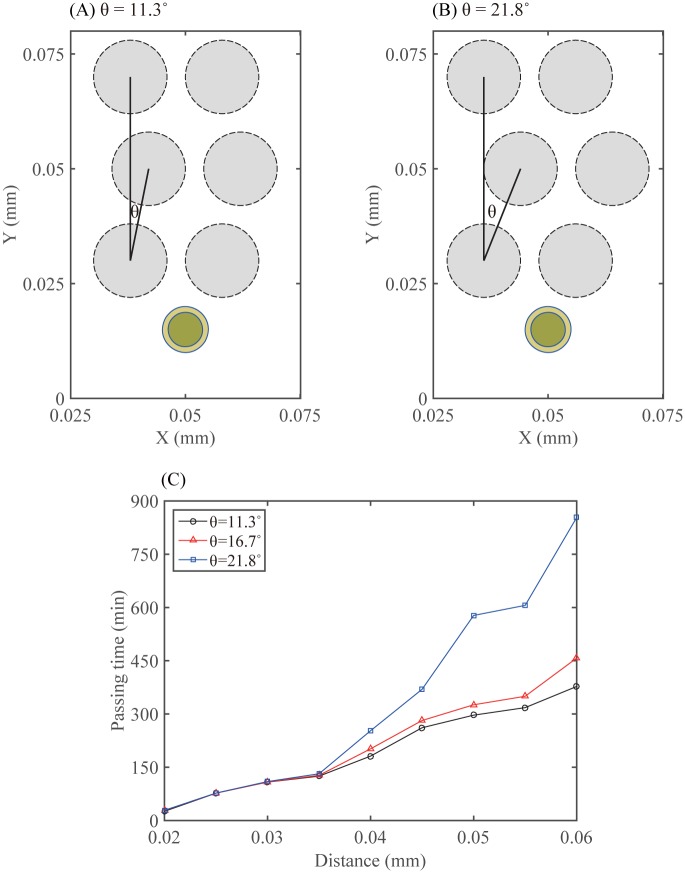
Analysis of passing time in response to microenvironmental complexity. (A,B) Initial configurations of six normal glial cells (dotted circle) and a migratory glioma cell (double circles) when the turning angles are given as *θ* = 11.3° (A) and *θ* = 21.8° (B), respectively. Here, *θ* = angle between two vectors connecting centers of two static normal glial cells: one for cells in the first and third rows, and another for cells in the first and second rows. The distance between two cells in each row is fixed. (C) Time at which a migratory glioma cell travels given distances (x-axis) under various degrees of complexities of normal cells (*θ* = 11.3° (empty circle), 16.7° (triangle), 21.8° (square)).

Positive values correspond to the cell movement in the north-east direction and negative values correspond to the cell movement in the north-west direction. In Fig 18 we investigate the effect of the complexity degree in an interstitial space between normal glial cells on cancer cell infiltration. Fig 18A and 18B show the initial configurations of six normal glial cells (dashed circles) and a migratory glioma cell (solid double circles) when turning angles are given as *θ* = 11.3° (Fig 18A) and *θ* = 21.8° (Fig 18B), respectively. Here, *θ* is the angle between two vectors connecting centers of two static normal glial cells: one for cells in the first and third rows, and another for cells in the first and second rows. The distance between two cells in each row is kept the same. Fig 18C shows the time (*min*) at which a migratory glioma cell travels given distances (x-axis) under various degrees of complexities of microenvironment (*θ* = 11.3° (empty circle), 16.7° (triangle), 21.8° (square)). The invasive cancer cell has to spend more time in order to infiltrate the network of normal cells as the distortion degree (*θ*) of the intercellular gap is increased (*θ* = 11.3° → *θ* = 21.8°).

**Fig 19:**
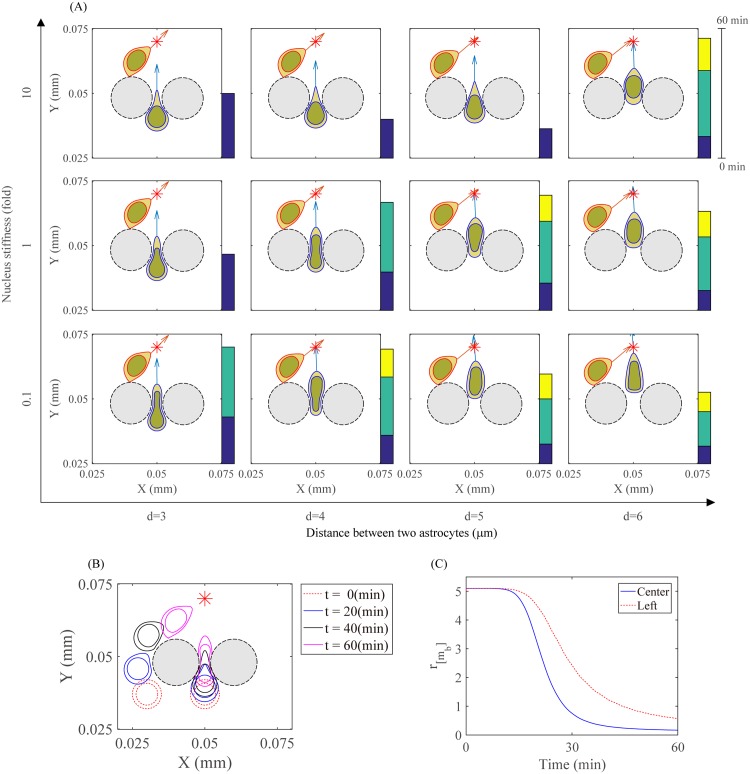
Patterns of glioma infiltration in different intra- and extra-cellular microenvironment. (A) Different patterns of two migratory glioma cells at final time t = 60 *min* for various distances between two astrocytes (*d* = 3, 4, 5, 6 *μm*) and different nuclear stiffness (fold) of a glioma cell located in the center. The default parameter value of the elastic stiffness of nucleus is $c_{\text{e}}^{Gn,b}=3.8\times 10^{-5}g\cdot cm/s^{2}$. (B,C) A migration profile and stiffening rate (*r*_[*m*_*b*_]_) of two migratory glioma cells at time *t* = 0, 20, 40, 60 mins when *d* = 4*μm* and nuclear stiffness is a fold-change of 1 for the migratory glioma cell in the center. The default parameter value was used for the glioma cell on the left.

How physical properties and structures of extracellular components in brain microenvironment control glioma progression is not completely understood [66–68]. The typical GBM tissue consists of tumor cells, microglia (or macrophages), which occupies up to 30% of the tissue [68], and other cell types such as astrocytes [69]. Astrocytes can play a significant role in regulation of primary and secondary CNS tumors [70, 71] and also form a secondary wall in blocking glioma invasion or penetrate the GBM in response to various CSPG levels [69, 72]. Glioma cells may adapt to different physical environments in various ways for their infiltration. Fig 19A shows migration patterns of two glioma cells in the different microenvironment captured at time *t* = 60 *min*. Twelve cases were examined for various distances between two astrocytes (*d* = 3, 4, 5, 6*μm*) and different nuclear stiffness (fold = 0.1, 1, 10) of a glioma cell located in the center relative to the basic stiffness parameter $c_{\text{e}}^{Gn,b}$ in Eq (22). The initial positions of two astrocytes and two glioma cells were marked as two gray-filled circles and two red-dashed concentric circles, respectivley, in Fig 19B. The color bar on the right of each sub-pannel in Fig 19A indicates time durations spent before entering a narrow gap (blue, *Y* = 0.04 *mm*), during the passage of the narrow intercellular space (green, *Y* = 0.05 *mm*), and after passing the gap (yellow, *Y* = 0.06 *mm*). As the distance between two astrocytes increases, the glioma cell in the center tends to pass through the intercellular gap easily. However, the glioma cell with much stiffer nucleus slows down the infiltration even when the distance between the two cells is wide enough (for example, *d* = 5*μm*, 10 fold). When the gap is too narrow (*d* = 3*μm*), the glioma cell in the center is unable to pass through even when the nuclear stiffness is low (0.1 fold). One can observe that the glioma cell starting initially on the lower left corner of the domain in all twelve cases can bypass the small narrow intercellular space and migrate around the left side of the astrocyte without nuclear deformation, which minimizes energy. Fig 19B, in particular, shows a profile of glioma cells at times *t* = 0, 20, 40, 60 *min* when *d* = 4*μm* and nucleus stiffness of the glioma cell on the center is a fold-change of 1. Fig 19C shows the stiffening rate (*r*_[*m*_*b*_]_) of the two migratory glioma cells corresponding to Fig 19B. The stiffening rate of the glioma cell in the center (solid blue line) drops down quickly while *r*_[*m*_*b*_]_ of the glioma cell on the left (dotted red line) is relaxed slowly. This suggests that the cancer cell infiltration in GBM and/or on the periphery of the tumor mass depends on spatial arrangement of cancer cells and other types of cells such as astrocytes. For example, tumor cells with different nuclear stiffness may adapt to the relative structure of astrocytes in the tumor mass for their optimal infiltration through astrocytes. The presence of CSPG in the tumor region may affect the physical condition of astrocytes, microglia, and tumor cells [69, 72]. A high level of CSPG tends to activate microglia within the tumor and induce accumulation of astrycotes on the periphery, leading to a non-invasive tumor, while astrocytes are distributed throughout the invasive tumor mass in the absence of CSPG [69]. This physical arrangement of astrocytes and microglia affects glioma cell infiltration. However, the basic mechanism for this phenomenon is not clearly understood. Our work indicates that the optimized infiltration strategy of glioma cells in response to regional heterogeneity in the distribution of brain astrocytes [71, 73] and microglia [69] as well as cellular response to intrinsic nuclear stiffness of heterogenous glioma cells in a given position might lead to totally different tumors: either non-invasive or invasive tumor.

**Fig 20:**
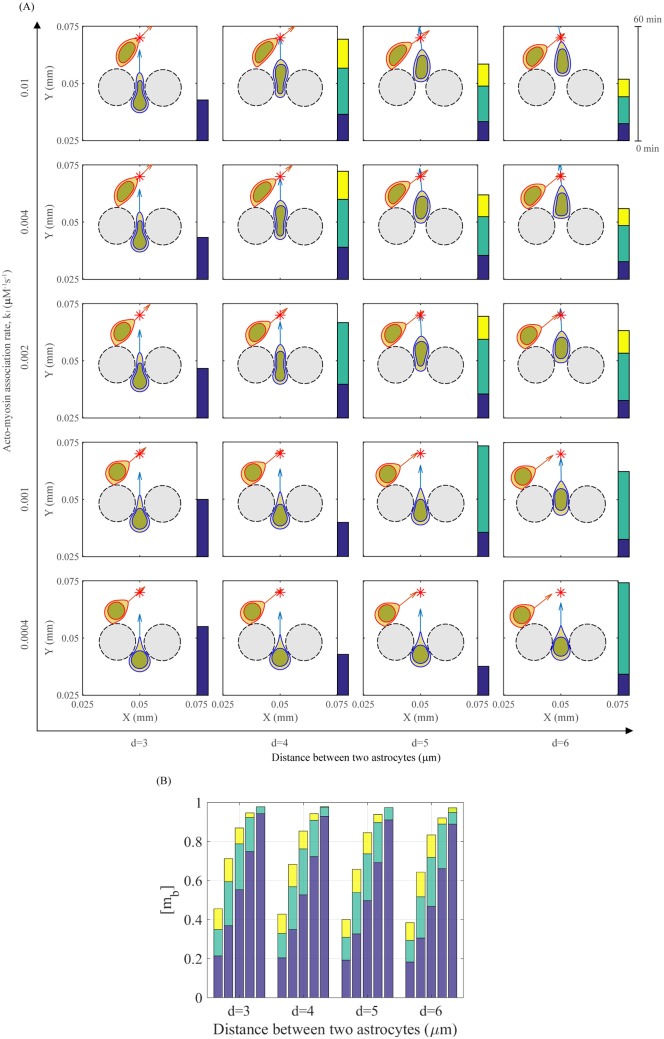
Patterns of glioma infiltration under perturbation of actin-myosin reactions and different microenvironment. (A) Different patterns of two migratory glioma cells at final time t = 60 *min* for various distances between two astrocytes (*d* = 3, 4, 5, 6 *μm*) and acto-myosin association rates (*k*_1_ = 0.0004, 0.001, 0.002, 0.004, 0.01 *μM*^−1^ *s*^−1^). (B) Distribution of the bound myosin II level ([*m*_*b*_]) of the glioma cell for different *k*_1_ and various distances *d* = 3, 4, 5, 6 *μm* simulated in (A). Each color indicates the change in concentration of the bound myosin II for 20 minutes.

Fig 20A shows patterns of two glioma cells for various distances (*d* = 3, 4, 5, 6 *μm*) between two astrocytes and acto-myosin association rate (*k*_1_ = 0.0004, 0.001, 0.002, 0.004, 0.01 *μM*^−1^ *s*^−1^). Cells were initially positioned at the same location as in Fig 19. For a fixed *k*_1_, an increase in *d* leads to higher probability of glioma cell infiltration. On the other hand, a decrease in acto-myosin association rate, while the distance (*d*) being kept the constant, can block the glioma infiltration through the narrow gap between two cells. This implies that the glioma invasion may be blocked by interfering the binding between actin components and free myosin II. Fig 20B shows the distribution of the bound myosin II level ([*m*_*b*_]) of the infiltrating glioma cell for different *k*_1_ and various distances *d* = 3, 4, 5, 6 *μm* given in Fig 20A. Each color indicates the change in concentration of the bound myosin II for 20 minutes.

**Fig 21:**
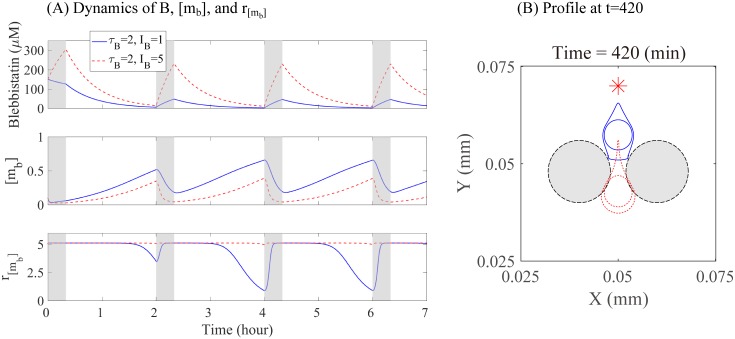
Therapeutic strategies: inhibition of tumor infiltration using blebbistatin. (A) Time courses of blebbistatin level, concentrations of bound myosin II ([*m*_*b*_]), and stiffening rate of nucleus (*r*_[*m*_*b*_]_) in response to blebbistatin injection with two different doses (*τ*_*B*_ = 2, *I*_*B*_ = 1 and *τ*_*B*_ = 2, *I*_*B*_ = 5). (B) Profile of a glioma cell in the presence of blebbistatin injection with *τ*_*B*_ = 2, *I*_*B*_ = 1 (blue solid curve) and *τ*_*B*_ = 2, *I*_*B*_ = 5 (red dotted curve). The relatively low dose of blebbistatin (*I*_*B*_ = 1) cannot sufficiently decrease the bound myosin II level and hence the stiffening rate of the nucleus is lowered, resulting in invasion of the glioma cell through the narrow gap. When the injection strength is increased (*I*_*B*_ = 5), the glioma cell cannot infiltrate the narrow intercellular space between two normal cells.

We now develop therapeutic strategies using anti-invasion drugs that target myosin II. Blebbistatin, a cell-permeable suppressor of class-II myosins, was developed to study the biologic roles of myosin II [74] and inhibition of glioma invasion [75, 76] due to its high affinity and selectivity toward myosin II [77]. For example, *in vitro* and *in situ* studies suggested that blocking migration of C6 glioma cells by directly targeting myosin II with blebbistatin was very effective even in the presence of several effective signaling pathways such as EGF and PDGF [76]. While ligands such as EGF, HGF, and PDGF can stimulate glioma invasion, blocking of these signaling pathways by anti-cancer drugs has not been so effective [78, 79] due to signal transduction redundancy in response to these drugs and co-expression of these molecules [80–84]. Thus, the development of optimal injection strategies of myosin II-targetting drugs such as blebbistatin can be an effective glioma treatment option since the myosin II is a point of convergence of many signaling networks regardless of how many upstream signal transduction cascades are active [76]. The name, blebbistatin, was originated from its ability to block cell membrane blebbing [85]. Blebbistatin inhibits the activity of myosin II by capping the bound myosin instead of interfering the actin-myosin binding [74]. We introduce a new equation for blebbistatin dynamics (*B*(*t*)) and the kinetic equation for bound myosin II [*m*_*b*_] is changed as follows

$$\begin{array}{c} \frac{dB}{dt}=\sum_{j}I_{B}\left(H_{t_{j}}-H_{t_{j}+\frac{1}{3}}\right)-\mu_{B}B, \end{array}$$ (30)

$$\begin{array}{c} \frac{d[m_{b}]}{dt}=k_{1}\left[m_{T}\right]\left[a\right]-\left(k_{1}\left[a\right]+k_{-1}\right)\left[m_{b}\right]-\alpha B\left[m_{b}\right], \end{array}$$ (31)

where *I*_*B*_ is the injection strength and *H* is a heaviside function, in which the injection is given for the first $\frac{1}{3}$ hour of every cycle $\tau_{B}=t_{j+1}-t_{j}\;\left(\geq \frac{1}{3}\;hour\right),\;j=0,\cdots ,N_{B}-1$, where *N*_*B*_ is the total number of blebbistatin injection and *t*_0_ = 0. *μ*_*B*_ is the decay rate of blebbistatin, and *α* is the consumption rate of the bound myosin isoform by blebbistatin. Initial condition was set to be 150 *μM* [77]. Fig 21 shows the dynamics of the acto-myosin system and biomechanical infiltration of glioma cells through two normal glial cells in response to two different doses of blebbistatin (*I*_*B*_ = 1, 5) with a fixed dose schedule (*τ*_*B*_ = 2 *hour*). When the blebbistatin dose is relatively low (*I*_*B*_ = 1), the level of bound myosin II is still relatively high and the stiffening rate of nucleus decreases (blue solid curves in Fig 21A), resulting in infiltration of the glioma cell (Fig 21B). When this dose level is increased to *I*_*B*_ = 5, the bound myosin II activity ([*m*_*b*_]) is decreased and the stiffening rate stays at the high level (red dotted curves in Fig 21A), resulting in inhibition of infiltration of the glioma cell through the narrow gap (Fig 21B).

**Fig 22:**
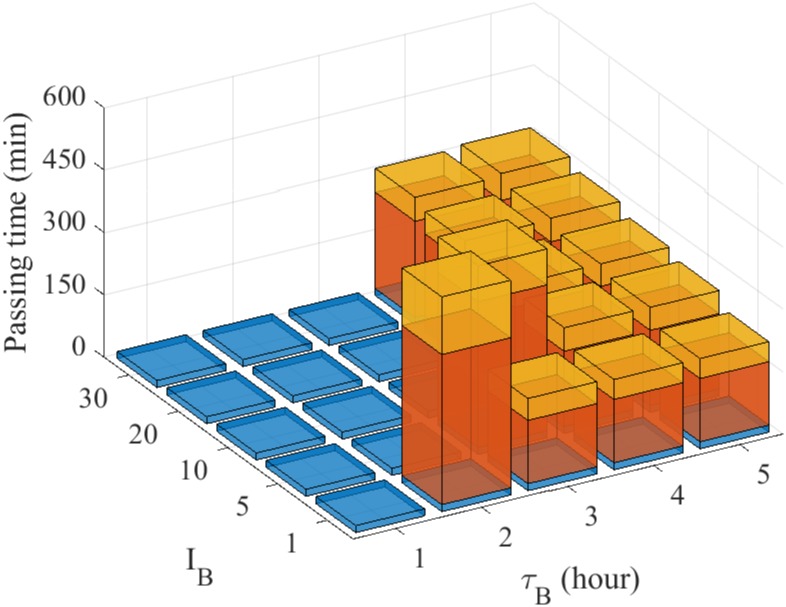
Optimal anti-invasion strategies of blebbistatin injection. Passing time of the glioma cell through the intercellular space between two glial cells for various dose schedules (*τ*_*B*_ = 1, 2, 3, 4, 5 *hours*) and injection strength (*I*_*B*_ = 1, 5, 10, 20, 30). *Blue = non-invasive glioma cell, red = the glioma cell in the process of infiltration through the gap, yellow = complete infiltration of the cell.

In Fig 22, we show passing time of a glioma cell through the intercellular space between two glial cells for various dose strength (*I*_*B*_ = 1, 5, 10, 20, 30) and schedules (*τ*_*B*_ = 1, 2, 3, 4, 5) of blebbistatin injection. For a fixed value of *I*_*B*_, the low dosing frequency (high *τ*_*B*_) induces higher accumulation of the bound myosin II and thus infiltration of the glioma cell. For a fixed dose frequency, the system switches from the migratory phase to the non-invasive phase as the injection dose amount (*I*_*B*_) is increased as shown in Fig 21. The model predicts that the larger injection interval (*τ*_*B*_) and smaller dose would lead to cell infiltration while the smaller injection interval and larger injection strength would block cell migration through the narrow gap. Even though blebbistatin’s side effects and toxicity on ion channels are relatively small compared to other uncouplers, precautions for perfusion with blebbistatin need to be taken [86]. Therefore, one would want to optimize the dose schedules in order to minimize the dose amount while still blocking the glioma infiltration. On the other hand, too frequent injection may not be either feasible or desirable due to administrative constraints at a clinic. In our simulations, the optimal treatment would be obtained when *τ*_*B*_ = 2, *I*_*B*_ = 5 in Fig 22.

**Fig 23:**
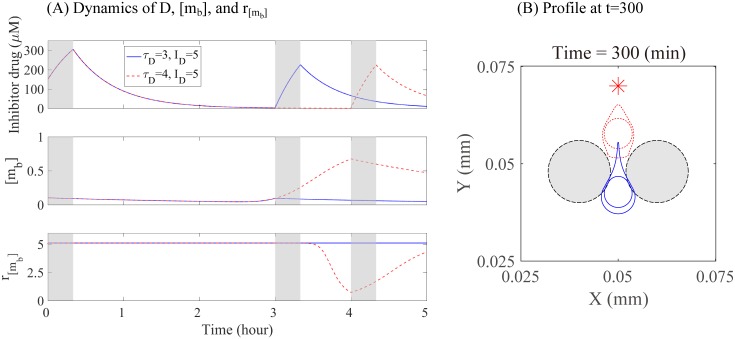
Therapeutic strategies: Inhibition of the activity of actin-myosin reactions. (A) Time courses of drug concentration, bound myosin II level ([*m*_*b*_]), and stiffening rate of nucleus (*r*_[*m*_*b*_]_) in response to inhibitor injection with two different schedules (*τ*_*D*_ = 3, *I*_*D*_ = 5 and *τ*_*D*_ = 4, *I*_*D*_ = 5). (B) Profile of a glioma cell in response to inhibitor injection with *τ*_*D*_ = 3, *I*_*D*_ = 5 (blue solid curve) and *τ*_*D*_ = 4, *I*_*D*_ = 5 (red dotted curve). The frequent dose of inhibitor (*τ*_*D*_ = 3) can keep the bound myosin II level low and maintain the stiff nucleus, resulting in inhibition of cell infiltration (blue). When this dose schedule is relaxed to *τ*_*D*_ = 4, the inhibitor drug is unable to suppress the accumulation of the bound myosin II, leading to flexible nucleus and glioma cell infiltration.

We now investigate the effect of drugs that inhibit the activity of myosin II by interfering the reaction between actin and myosin II. We introduce a new equation for this dynamics (*D*(*t*)) and the kinetic equation of bound myosin II [*m*_*b*_] is changed as follows

$$\begin{array}{c} \frac{dD}{dt}=\sum_{j}I_{D}\left(H_{t_{j}}-H_{t_{j}+\frac{1}{3}}\right)-\mu_{D}D, \end{array}$$ (32)

$$\begin{array}{c} \frac{d[m_{b}]}{dt}=k_{1}e^{-D}\left[m_{T}\right]\left[a\right]-\left(k_{1}e^{-D}\left[a\right]+k_{-1}\right)\left[m_{b}\right]. \end{array}$$ (33)

where *I*_*D*_ is the injection strength, $\tau_{D}=t_{j+1}-t_{j}\;\left(\geq \frac{1}{3}\;hour\right),\;j=0,\cdots ,N_{D}-1$, where *N*_*D*_ is the total number of drug injection, *μ*_*D*_ is the decay rate of the drug. As before, the injection is given for the first $\frac{1}{3}$ hour of each *τ*_*D*_. In Fig 23, we illustrate two different patterns of passing and non-passing gliomas in response to two different time schedules (*τ*_*D*_ = 3, 4 *hours*) with the fixed drug strength (*I*_*D*_ = 5). The glioma cell passes through the narrow intercellular space between two glial cells for the longer time interval between drug injections unlike the case with the shorter time interval. Thus, given injection amount (*I*_*D*_), one can inhibit the infiltration of a glioma cell by decreasing the injection interval (*τ*_*D*_) in order to switch the motility phase from a passing state to the non-passing state.

**Fig 24:**
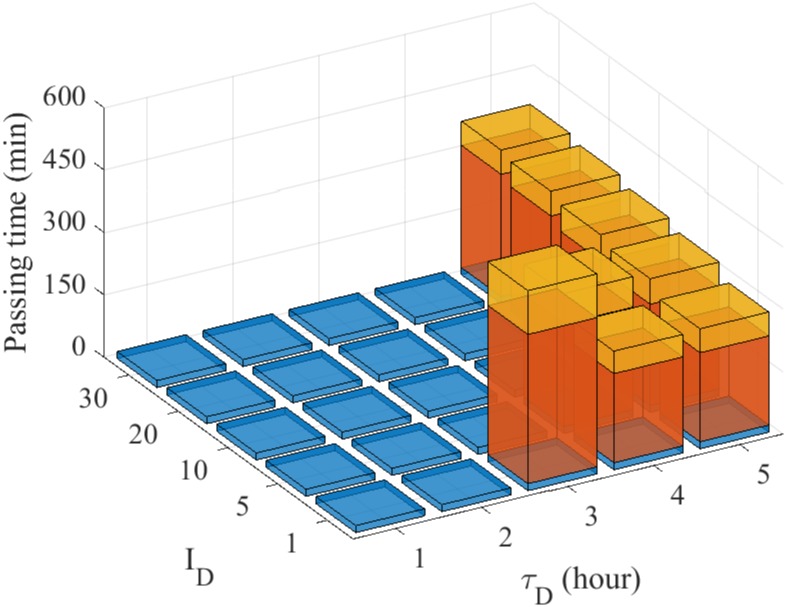
Optimal anti-invasion strategies for drugs that inhibit the activity of actin-myosin reactions. Passing time of the glioma cell through the intercellular space between two glial cells for various dose schedules (*τ*_*D*_ = 1, 2, 3, 4, 5 *hours*) and injection strength (*I*_*D*_ = 1, 5, 10, 20, 30). *Blue = non-invasive glioma cell, red = the glioma cell in the process of infiltration through the gap, yellow = complete infiltration of the cell.

In Fig 24, we develop an optimal strategy of drug injection by investigating the infiltration potential of a glioma cell through the intercellular space between two glial cells for various dose strength (*I*_*D*_ = 1, 5, 10, 20, 30) and schedules (*τ*_*D*_ = 1, 2, 3, 4, 5). For a fixed dose of *I*_*D*_, the longer interval of *τ*_*D*_ keeps the accumulation of the bound myosin II at the higher level, which leads to infiltration of the glioma cell. For a fixed dose frequency, the cell switches from the infiltration to the non-invasive phase as the injection strength (*I*_*D*_) is increased as shown in Fig 24. The model predicts that the frequent high dose of the inhibitory drugs would be necessary in order to inhibit cell infiltration through the narrow gap. Similarly, glioma cell invasion can be blocked by increasing injection strength (*I*_*D*_) of the drug, while the injection interval is fixed. Based on these observations, the combination of *τ*_*D*_ = 3 and *I*_*D*_ = 5 would be an optimal choice to reduce administrative expenses related to the injection process at a hospital as well as to avoid possible side effects from high dose drugs.

## Discussion

Cell migration has two different aspects: (i) it is necessary for numerous physiological processes such as wound healing and immune responses to pathogens, and (ii) it can lead to metastasis of cancer cells. A main reason for treatment failure of glioblastoma is that by the time the disease is diagnosed cancer cells have already invaded other parts of the brain [6]. In order to block, reverse or even counterattack this critical invasion process, one ought to understand the fundamental regulation of this cell migration. It is therefore important to investigate the biochemical regulation of cell invasion [12] and find a novel way of blocking [6, 87] or controlling [15, 16] the migratory behavior of glioma cells through a dense network of normal cells in the microenvironment. Glioma cell migration in patients is a multistep process that effectively uses all the necessary components such as the cytoskeleton, extracellular matrix, integrins, signaling networks, proteases, and ion channels [59]. Many of these elements have been considered as therapeutic targets for the development of anti-invasion strategies. However, agents that target these invasive components have shown limited clinical outcomes [88], which requires alternative anti-invasive strategies. A more comprehensive understanding of how glioma cells interact with [72, 89] and infiltrate within the complex microenvironment of the brain would be a prerequisite for the development of a new effective anti-cancer treatment.

In this paper we developed a mathematical model to understand the regulation of cell migration through the densely packed neuropil of the brain, to investigate cell mechanics in regulating invasion patterns under the influence of various environmental factors such as chemoattractants and physical/mechanical constraints, and to lay down a general framework of the cell motility for developing optimal strategies of blocking glioma invasion. The full multi-scale model considers the movement of a glioma cell under mechanical constraints in heterogeneous cell tissue based on the cell mechanics, the concentration of chemoattractants, and the acto-myosin dynamics in the spatio-temporal domain. Mechanical stresses and active forces acting on each cell were taken into account in the model and these features allowed us to explore the effect of cell mechanics on the cell motility.

Cell motility requires the formation of cytoplasmic contractile force. Hence, one way of examining how glioma cells invade into brain is to investigate how these cells utilize myosin, the major source of cytoplasmic contractile force. For example, fibroblasts and carcinoma cells use myosin II to drive contraction of the cell posterior, to disconnect the motile cell from its extracellular matrix attachments, to expand the leading lamellipodium, and to generate and maintain the cell polarity [90, 91]. By contrast, myosin II plays a different role in neural progenitor cells, where it seems to be involved in the regulation of translocation of the nucleus [10]. It is not clear whether these different migration mechanisms between fibroblasts and neural progenitor cells are induced from intrinsic differences between non-CNS-derived and CNS-derived migratory cells, or whether these cells adapt to the different microenvironment for their migration. While glioma cell migration involves the extension of a broad lamellipodium and the continuous movement without nuclear distortion in the absence of spatial constraints, they move in a manner similar to neural progenitor cells [10] when faced migration through the white matter [11].

The mechanical contribution of motor proteins is controlled by a complex intracellular biochemical signaling network in response to external stimuli. Such biomechanical adaptation is necessary for cells to migrate through a microenvironment where a round-shaped nucleus hinders the deformation of the cell passing through a narrow gap. Beadle *et al.* [11] found experimentally that the glioma cell infiltrates the narrow intercellular gap in a dense network of normal brain glial cells by deforming the nucleus via up-regulation of myosin II, however, the cancer cells cannot migrate through the narrow space between normal cells when the myosin II is knocked down. This suggests that myosin II is required for migration through small pores smaller than the diameter of the nucleus. Our simulation results showed that active migration of a glioma cell in brain tissue depends on the myosin II concentration in response to chemoattractants and its physical microenvironment. In our model, the migratory glioma cells in wild type were able to migrate through the narrow extracellular gap, whereas cell migration in MYOII-KD case was blocked. Cell speeds in wild type and MYOII-KD were in good agreement with experimental data [9, 11]. Glioma cells may adapt to the harsh microenvironment, the submicrometer size of the extracellular spaces between the tightly packed neuropil of the brain [92], by using myosin II so that the cells modify their shape and pass through the narrow gap, instead of using a lamellipodium [11]. Cancerous cells often adopt the amoeboid migration through a narrow gap bordered by fibers or after abrogation of pericellular proteolysis [93]. Our results demonstrated that the coordination of biochemical and mechanical components within the glioma cell plays an important role in amoeboid migration [87] for adaptations to narrow intercellular gaps with small pore sizes [11]. We also showed that a major role of myosin II is to push the bulky nucleus and cell body through the narrow gaps within the brain matrix for this amoeboid movement. It is known that cells can go through transitions between mesenchymal and amoeboid migration. Cortical contractility and flow, for example, can trigger a stochastic switch from mesenchymal and blabbing modes to prototypic amoeboid migration mode, forming stable-bleb in the confined microenvironment [94]. In other cells such as dendritic cells, perinuclear Arp2/3-driven actin polymerization, not myosin II, was shown to play a central role in nuclear deformation for cell migration through narrow gaps in a complex environment [95]. What makes these differences in adapting mesenchymal machinary, myosin II- or actin-driven motors, or blabbing, among cell types is not completely understood. Further investigation on exact balance of myosin II and accumulation of actin networks near the nucleus is necessary for the deep understanding of the nucleus deformation and cell infiltration.

The control of the regrowth of invasive cells after or before surgery might lead to better therapeutic strategies [16–19]. Since more than 90% of glioma recurrence occurs within 2 *cm* from the primary glioma site [96], the anti-cancer invasion strategy by injecting the chemoattractant on the periphery of the resection site may be effective for eradication of these infiltrative cells in the local area. But such strategies still have a distance limitation [18]. For example, residual enhancement of 1-year post-G207 inoculation (oncolytic virus) is decreased in the area close to the resection site in human glioma patient, but new enhancement of the tumor can emerge on the opposite side of the brain in the contralateral hemisphere [97]. The efficacy of anti-invasion strategies [4, 6, 7] may depend on the physical structure of the brain tissue [9], alignment of normal cells [11], the ECM geometry [28], blood vessels [9], and microglia/astrocytes [69] as well as important intracellular Rho and Rac signaling which affects the protrusion, contraction, and proteolytic activities [98, 99]. In this work, we investigated how the glioma infiltration can be adapted in response to the physical arrangements of glioma cells with other cell such as astrocytes and microglia as well as physiological conditions such as nuclear stiffness and biochemical conditions of acto-myosin machinery. We also developed anti-invasion therapies by either interfering with binding between the actin components and myosin II or capping the bound myosin II by blebbistatin or drugs. Mutual interaction between glioma cells with other cells can affect its decision on cell infiltration in the network of extracellular matrix and various types of cells in the tumor microenvironment. We conclude that the anti-invasion therapy by targeting the merging point of all upstream networks, acto-myosin component, can be effective in a well conditioned environment but it may have to be well-designed in order to overcome various adaptation the cancer cell may have to in the heterogeneous conditions [68, 69, 71, 73]. These provide us a foundation for anti-invasion or local therapies [100]. A more detailed model with the control of cell-cell adhesion [4], more realistic cell cortex and nuclear envelope [27], and other microenvironmental factors listed above may shed lights on how to develop drugs that selectively target these invasive cells [101] among other normal cells in the brain.

This work provides a general framework for multi-step processes of cell migration due to different affinities of glioma cells toward particular components such as myelin tracks [102–104]. In addition, our simulation results envision the development of anti-invasion therapies by targeting signaling networks involving integrin receptors, cytoplasmic adhesion molecules, and actin cytoskeletal dynamics [87]. The results of this paper serve as a starting point for more detailed modeling and experimentation. In this study, we did not focus on cell migration in the perivascular space. Obviously, migration of glioma cells in the perivascular space of Virchow-Robin (which is essentially fluid-filled space) [105, 106] is different from migration along white matter tracts in the neuropil, where glioma cells indeed need to squeeze themselves through. Our understanding of the delicate biochemical/mechanical interactions between a glioma cell and the microenvironment is very limited. Yet a more comprehensive understanding of the role of the microenvironment in glioma cell invasion [89] may lead to the development of new therapeutic drugs that target these stromal elements and glioma stem cells [101] in addition to tumor cells. We plan to address these issues in future work.

## Supporting information

S1 File Permission from publisher. Permission for Figs 1(left), 2, 7(A)–7(C), 9F was obtained from Beadle C, Assanah M, Monzo P, Vallee R, Roseneld S, etal. (2008) The role of myosin II in glioma invasion of the brain. Mol Biol Cell 19:3357-3368 under a CC BY license.
(PDF)
