# Supplementary material for: The role of myosin II in glioma invasion: A mathematical model
Source: PLoS One. 2017 Feb 6;12(2):e0171312. doi: 10.1371/journal.pone.0171312 (PMC5293275; doi:10.1371/journal.pone.0171312)
Supplement: S1 File — Permission for Figs 1(left), 2, 7(A)–7(C), 9F was obtained from Beadle C, Assanah M, Monzo P, Vallee R, Roseneld S, etal. (2008) The role of myosin II in glioma invasion of the brain. Mol Biol Cell 19:3357-3368 under a CC BY license. (PDF) [file pone.0171312.s001.pdf]

[seeking permission of Figures in an article in Mol Biol Cell]

ASCB MBC <MBC@ascb.org> Fri, Dec 23, 2016 at 12:59 AM
To: Yangjin Kim <ahyouhappy@gmail.com>, ASCB MBC <MBC@ascb.org>

Dear Dr. Kim,

Permission is granted to reproduce Fig 1, Fig 4A, Fig 5, and Fig 1 in supplementary material in Beadle C, Assanah M, Monzo P, Vallee R, Roseneld S, et al. (2008) The role of myosin II in glioma invasion of the brain. Mol Biol Cell 19: 3357-3368 under a CC BY license provided that you cite the original source.

Best regards,

W. Mark Leader

Publications Director

Editor, ASCB Newsletter

American Society for Cell Biology

8120 Woodmont Ave., Suite 750

Bethesda, MD 20814

mleader@ascb.org

301-347-9317

From: Yangjin Kim [mailto:ahyouhappy@gmail.com]
Sent: Wednesday, December 21, 2016 9:27 PM
To: ASCB MBC
Cc: Yangjin Kim
Subject: [seeking permission of Figures in an article in Mol Biol Cell]

[Quoted text hidden]
